# Effects and possible mechanisms of action of acacetin on the behavior and eye morphology of *Drosophila* models of Alzheimer’s disease

**DOI:** 10.1038/srep16127

**Published:** 2015-11-04

**Authors:** Xue Wang, Haribalan Perumalsamy, Hyung Wook Kwon, Young-Eun Na, Young-Joon Ahn

**Affiliations:** 1 Department of Agricultural Biotechnology, Seoul National University, Seoul 151-921, Republic of Korea; 2 Research Institute of Agriculture and Life Sciences, Seoul National University, Seoul 151-921, Republic of Korea; 3 R&D Coordination Division, Rural Development Administration, Jeonju560-500, Republic of Korea; 4 College of Plant Science & Technology, Huazhong Agricultural University, Wuhan 430070, Hubei, P.R. China

## Abstract

The human β-amyloid (Aβ) cleaving enzyme (BACE-1) is a target for Alzheimer’s disease (AD) treatments. This study was conducted to determine if acacetin extracted from the whole *Agastache rugosa* plant had anti-BACE-1 and behavioral activities in *Drosophila melanogaster* AD models and to determine acacetin’s mechanism of action. Acacetin (100, 300, and 500 μM) rescued amyloid precursor protein (APP)/BACE1-expressing flies and kept them from developing both eye morphology (dark deposits, ommatidial collapse and fusion, and the absence of ommatidial bristles) and behavioral (motor abnormalities) defects. The reverse transcription polymerase chain reaction analysis revealed that acacetin reduced both the human *APP* and *BACE-1* mRNA levels in the transgenic flies, suggesting that it plays an important role in the transcriptional regulation of human *BACE-1* and *APP*. Western blot analysis revealed that acacetin reduced Aβ production by interfering with BACE-1 activity and APP synthesis, resulting in a decrease in the levels of the APP carboxy-terminal fragments and the APP intracellular domain. Therefore, the protective effect of acacetin on Aβ production is mediated by transcriptional regulation of *BACE-1* and *APP*, resulting in decreased APP protein expression and BACE-1 activity. Acacetin also inhibited APP synthesis, resulting in a decrease in the number of amyloid plaques.

Alzheimer’s disease (AD) is a neurodegenerative disorder and the most prevalent form of dementia in developed and developing countries. Worldwide, the number of people with dementia was estimated to be 35.6 million in 2010; this figure is expected to reach 115.4 million by 2050^1^. The worldwide societal cost of dementia, including direct and indirect care, was an estimated 604 billion USD in 2010, an increase of 288.6 billion from 315.4 billion USD in 2005^2^. Fourteen and 40% of the people in low- and middle-income countries have dementia, respectively, which accounts for less than 1% and 10% of the total worldwide costs, respectively. However, 46% of the people in high-income countries have dementia, accounting for 89% of the costs^2^. AD accounts for 60–70% of dementia cases^3^,^4^. The major histopathological hallmarks of AD are neurofibrillary tangles composed of hyperphosphorylated tau protein filaments, and extracellular senile plaques, which are deposits of β-amyloid (Aβ) generated via sequential proteolytic processing of the transmembrane amyloid precursor protein (APP) by two enzymes in the amyloidogenic processing pathway, namely β-secretase (β-site APP cleaving enzyme or BACE-1) and γ-secretase^5^,^6^,^7^.

AD is currently treated using acetylcholinesterase (AChE) inhibitors^8^ and *N*-methyl-D-aspartate (NMDA) receptor antagonists^9^,^10^. However, these treatments do not stop the disease process or prevent neuronal degeneration^11^. These treatments also have serious side effects^9^,^12^. BACE-1 is considered a primary target for preventing and treating AD^13^,^14^,^15^. Many peptidomimetics and heterocyclic compounds have been evaluated as BACE-1 inhibitors^16^,^17^,^18^; however, none of these have been successfully developed as anti-AD drugs. Therefore, there is a pressing need to develop new improved anti-AD agents.

Natural compounds from plant extracts have been suggested as alternative sources for anti-AD drugs. This approach is appealing, in part, because plants are sources of bioactive secondary metabolites that are perceived by the general public as relatively safe, and that often act on multiple, novel target sites^19^,^20^. Certain plant preparations and their constituents are regarded as potential sources for commercial anti-AD products to prevent or treat AD. Plant-derived BACE-1 and AChE inhibitors have been well documented by Orhan^18^ and Mukherjee *et al.*^21^, respectively. Previous studies have shown that a methanol extract from the whole blue licorice (Korean mint) plant, *Agastache rugosa* (Fisch. & C.A. Mey.) O. Kuntze (Lamiaceae), possessed human BACE-1 inhibitory activity. Historically, this plant species has been used to treat cholera, vomiting, and miasma^22^. However, no previous studies have investigated the potential use of *A. rugosa* for managing AD, although the phytochemistry and bioactivity of plants in the genus *Agastache* have been well documented by Zielinska and Matkowski^22^.

In this study, our aim was to assess whether the flavonoid, acacetin, and two triterpenoids, maslinic acid and oleanolic acid, extracted from the whole *A. rugosa* plant had BACE-1 inhibitory activity in comparison with commercial organic pure acacetin and two positive controls, the cell-permeable isophthalamide, BACE-1 inhibitor IV^23^, and the natural BACE-1 inhibitor, epigallocatechin gallate (EGCG)^24^, using a fluorescence resonance energy transfer (FRET)-based enzyme assay. The effects of the most potent inhibitory constituent, acacetin, on the eclosion rate, feeding, climbing, and life span of a *Drosophila melanogaster* mutant that co-expresses human *APP* and *BACE-1* within the developing nervous system were evaluated. In addition, the morphological changes in the compound eyes of the transgenic flies were examined using light microscopy and scanning electron microscopy (SEM). Finally, the possible mechanism underlying the anti-AD actions of acacetin was elucidated using real-time quantitative reverse transcription polymerase chain reaction (qRT-PCR) and western blot analyses.

## Results

### Fluorescence resonance energy transfer-based enzyme assay-guided fractionation and isolation

The fractions obtained from solvent partitioning of the methanol extract of the whole *A. rugosa* plant were tested for human BACE-1inhibitory activity using a FRET-based enzyme assay (Table 1). Significant differences in inhibitory activity were observed among the fractions and were used to identify the peak activity fractions for the next step of purification. At a concentration of 1 mg/mL, the hexane-soluble fraction was the most potent inhibitor, while no inhibition was obtained using the chloroform-, ethyl acetate-, butanol-, or water-soluble fractions.

**Figure 1:**
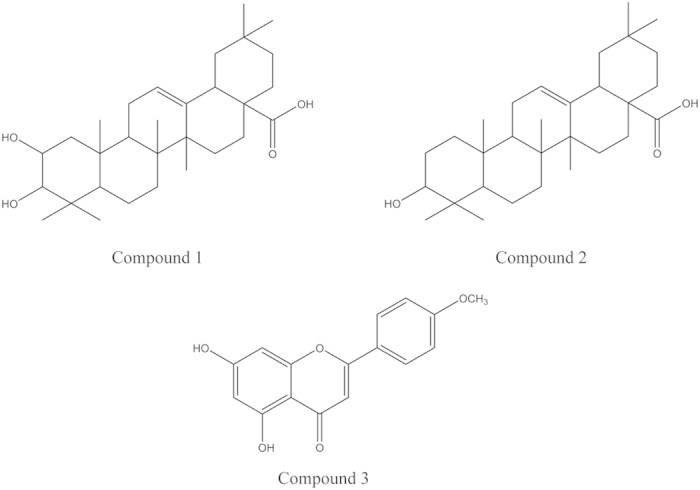
Structures of maslinic acid, oleanolic acid, and acacetin. These compounds were identified in the whole *Agastache rugosa* plants in this study. The chemical formula of maslinic acid (**1**) is C_30_H_48_O_4_, with a molar mass of 472.70 g/mol; the chemical formula of oleanolic acid (**2**) is C_30_H_48_O_3_, with a molar mass of 456.70 g/mol; and the chemical formula of acacetin (**3**) is C_16_H_12_O_5_, with a molar mass of 284.26 g/mol.

FRET-based enzyme assay-guided fractionation of the whole *A. rugosa* plant afforded three active compounds that were identified by spectroscopic analyses, including electron ionized mass spectrometry (EI-MS) and nuclear magnetic resonance (NMR) spectroscopy. The three BACE-1 inhibitory compounds were maslinic acid (**1**), oleanolic acid (**2**), and acacetin (**3**) (Fig. 1). Maslinic acid (**1**) was identified based on the following evidence: a white crystal; Ultraviolet (UV) (MeOH): λ_max_ nm = 217; EI-MS (70 eV), *m*/*z* (% relative intensity): 472 [M]^+^ (1.9), 284 (3.0), 256 (2.8), 248 (100), 233 (8.6), 203 (73.5), 189 (8.1), 173 (3.4), 133 (10.2), 105 (5.1), 95 (4.4), 69 (6.2), 55 (5.3) (see Supplementary Fig. S1 online); ^1^H NMR (MeOD, 600 MHz): δ 0.79 (3H, s), 0.86 (3H, s), 0.87 (3H, s), 0.94 (3H, s), 0.99 (3H, s), 1.10 (3H, s), 1.13 (3H, s), 2.89 (1H, m), 2.98 (1H, d, *J* = 9.54 Hz), 3.61 (1H, ddd, *J* = 4.50, 2.94, 3.90 Hz), 5.21 (1H, t, *J* = 6.72 Hz) (see Supplementary Fig. S2 online); and ^13^C NMR (MeOD, 150 MHz): δ 17.2 q, 17.6 q, 18.3 q, 19.8 t, 24.5 q, 24.7 q, 24.8 t, 26.6 t, 29.4 t, 29.5 q, 30.9 s, 31.9 q, 34.1 t, 34.2 t, 34.5 t, 39.5 s, 40.6 s, 40.7 s, 43.2 s, 43.8 d, 48.3 t, 48.4 s, 48.7 t, 49.0 d, 56.9 d, 69.7 d, 84.7 d, 122.5 d, 146.9 s, 185.8 s (see Supplementary Fig. S3 online). Oleanolic acid (**2**) was characterized as follows: a white amorphous powder; UV (MeOH): λ_max_ nm = 216; EI-MS (70 eV), *m/z* (% relative intensity): 456 [M]^+^ (4.3), 249 (18.8), 248 (100), 233 (6.0), 207 (17.3), 204 (11.3), 203 (59.7), 190 (10.0), 189 (10.6), 175 (7.4), 133 (15.0), 105 (6.7), 81 (6.1), 69 (8.0), 55 (7.6) (see Supplementary Fig. S4 online); ^1^H NMR (MeOD, 600 MHz): δ 0.77 (3H, s), 0.85 (3H, s), 0.90 (3H, s), 0.95 (3H, s), 0.97 (3H, s), 1.12 (3H, s), 1.15 (3H, s). 1.38 (2H, m), 1.41 (2H, m), 1.56 (4H, m), 2.20 (3H, m), 2.85 (1H, dd, *J* = 9.9, 4.5 Hz), 3.15 (1H, dd, *J* = 15.78, 4.4 Hz), 3.17 (1H, t, *J* = 14.76 Hz), 3.34 (2H, s), 4.62 (2H, s), 5.23 (1H, s) (see Supplementary Fig. S5 online); and ^13^C NMR (MeOD, 150 MHz): δ 16.0 q, 16.5 q, 16.5 q, 17.9 t, 21.7 t, 24.2 q, 24.3 t, 25.5 q, 26.5 t, 28.0 t, 28.9 q, 31.8 s, 33.8 t, 34.0 t, 34.2 q, 34.5 t, 35.1 s, 38.2 t, 38.3 s, 40.0 s, 40.7 d, 42.9 s, 43.0 s, 47.5 t, 47.9 d, 56.9 d, 79.8 d, 123.7 d, 145.5 s, 182.4 s (see Supplementary Fig. S6 online). Acacetin (**3**) was characterized as follows: yellow needles; UV (MeOH): λ_max_ nm = 269, 315; EI-MS (70 eV), *m/z* (% relative intensity): 284 [M]^+^ (100), 283 (12.5), 241 (11.4), 152 (6.9), 132 (18) (see Supplementary Fig. S7 online); High resolution EI-MS: C_16_H_12_O_5_ observed: 284.0683, calculated: 284.0684; Fourier transform infrared spectroscopy (FT-IR) ν_max_ cm^–1^: 3147 (-OH), 1651 (−C=O), 1605, 1560, 1503, 1428 (-C=C) (see Supplementary Fig. S8 online); ^1^H NMR (DMSO-*d*_6_, 600 MHz): δ 3.16 (3H, s), 6.20 (1H, d, *J* = 1.98 Hz), 6.51 (1H, d, *J* = 2.04 Hz), 6.87 (1H, s), 7.11 (2H, d, *J* = 8.88 Hz), 8.04 (2H, d, *J* = 8.88 Hz), 10.85 (1H, s), 12.92 (1H, s) (see Supplementary Fig. S9 online); and ^13^C NMR (DMSO-*d*_6_, 150 MHz): δ 55. 6 q, 94.0 d, 98.9 d, 103.5 d, 103.8 s, 114.6 d, 114.6 d, 122. 8 s, 128.3 d, 128.3 d, 157.3 s, 161.4 s, 162.3 s, 163.3 s, 164.2 s, 181.8 s (see Supplementary Fig. S10 online). The interpretations of the proton and carbon signals of compounds **1**, **2**, and **3** were largely consistent with those of Tanaka *et al.*^25^ and Dam *et al.*^26^, Hossain and Ismail^27^ and Gangwal *et al.*^28^, and Wawer and Zielinska^29^ and Miyazawa and Hisama^30^, respectively.

### *In vitro* BACE-1 inhibitory activity of the isolated compounds

The BACE-1 inhibitory activity of the three isolated compounds (acacetin, maslinic acid, and oleanolic acid), organic pure acacetin, and human BACE-1 inhibitor IV and EGCG, which were used as positive controls, were elucidated (Table 2). Based on the IC_50_ values, natural and pure organic acacetin had similar inhibitory activity, indicating that the activity of the methanol-extracted acacetin was purely due to acacetin. Natural acacetin was a 4.0-fold and 5.5-fold more potent inhibitor of BACE-1 than oleanolic acid and maslinic acid, respectively. The BACE-1 inhibitory activity of acacetin and EGCG did not differ significantly. Overall, these compounds were significantly less potent inhibitors of BACE-1 than BACE-1 inhibitor IV.

### Effect of acacetin on age-dependent neurodegeneration, as reflected by an aberrant eye phenotype

**Figure 2:**
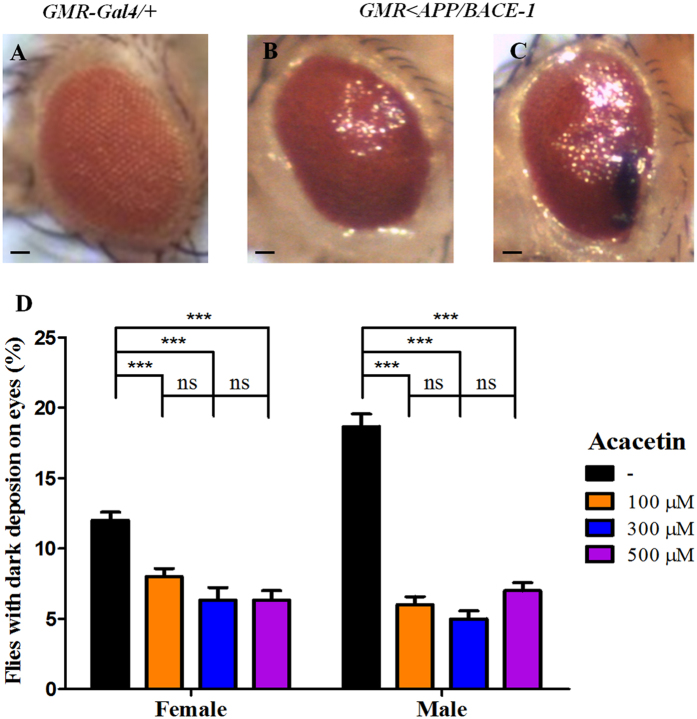
Effect of acacetin on the morphological defects in the compound eyes of the transgenic flies. Human APP and BACE-1 transgenic flies (*GMR* < *APP/BACE-1*) were cultured from the egg stage in polystyrene vials containing standard media supplemented with acacetin (100, 300, and 500 μM) in 0.1% DMSO. The morphology of the compound eyes of male and female flies (1 day old) was observed with a stereo microscope (×35). The flies with aberrant eye phenotypes were collected, and the number of flies with dark deposits in the eyes was counted. **(A)** Control male *GMR-Gal4* flies with a wild-type phenotype of a smooth surface compound eye. **(B)** Transgenic male *GMR* < *APP/BACE-1* flies with a compound eye with an abnormal appearance. **(C)** Male *GMR* < *APP/BACE-1* flies with morphological defects, particularly dark deposits in the eyes. **(D)** Quantitation of the phenotype of male and female flies showing serious morphological defects with dark deposits in the eyes. Each bar represents the mean ± SE from three independent experiments (^***^P < 0.001; ns, no significant difference, using Bonferroni’s multiple comparison test). The scale bars represent 20 μm.

Overexpression of human *BACE-1* and *APP* during *Drosophila* eye development resulted in an aberrant rough eye phenotype in the male *GMR* < *APP/BACE-1* flies (1-day-old) (Fig. 2B,C), particularly those with dark deposits in the eye (marked with an arrow in Fig. 2C), compared to the control male *GMR-GAL4*/+ flies with normal and well-organized compound eyes (Fig. 2A). Similar results were also observed with the transgenic female *GMR* < *APP/BACE-1* flies. The effects of acacetin on the morphological defects in the eyes with dark deposits were examined (Fig. 2D). Acacetin (100, 300, and 500 μM) suppressed the ratios of female flies (6.3–8.0%) and male flies (5.0–7.0%) with dark deposits in the eyes compared to the vehicle dimethyl sulfoxide (DMSO)-fed control females (12.0%) and males (18.7%).

**Figure 3:**
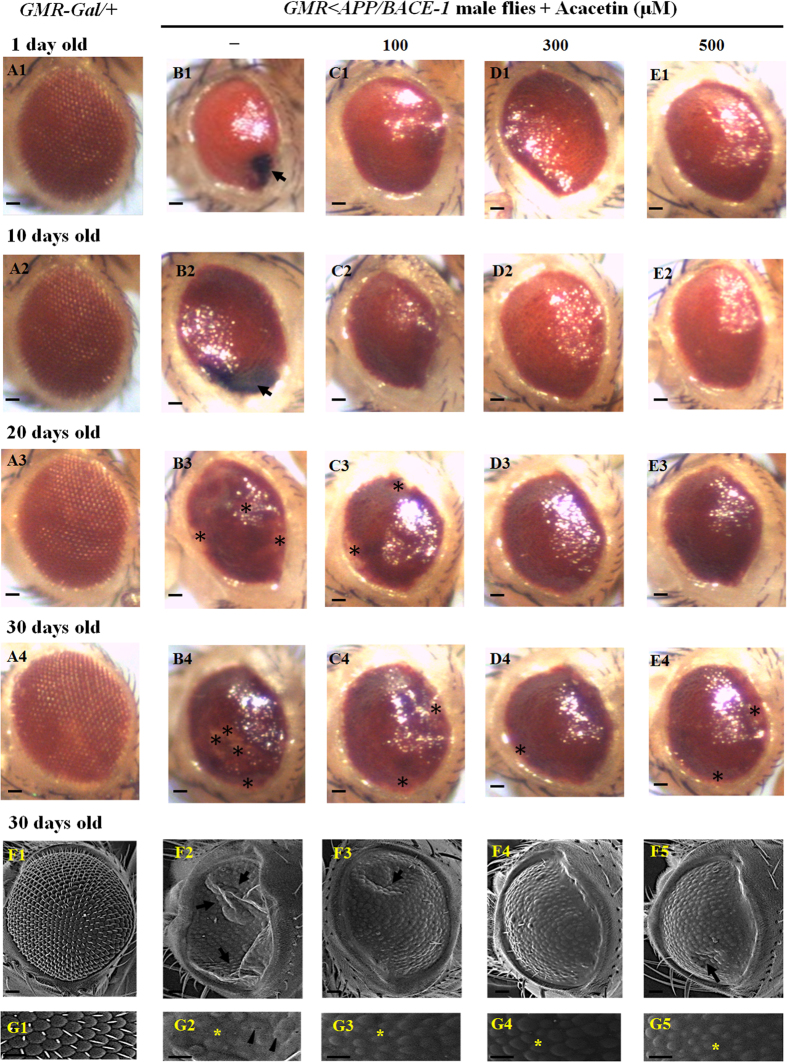
Effect of acacetin on the aberrant eye phenotype associated with age-dependent eye development. Human APP and BACE-1 transgenic flies (*GMR* < *APP/BACE-1*) were cultured from the egg stage in polystyrene vials containing standard media supplemented with acacetin (100, 300, and 500 μM) in 0.1% DMSO. Light micrographs. **(A)** The control male *GMR-GAL4*/+ flies (1, 10, 20, and 30 days old) had a normal and well-organized eye morphology. There were no obvious morphological changes as time elapsed. **(B)** The vehicle-fed male *GMR* < *APP/BACE-1* flies exhibited dark deposits at the edge of the compound eye, even at the youngest age evaluated (marked with an arrow in Fig. 3B1); the size of the deposits had increased at 10 days (marked with an arrow in Fig. 3B2). As time passed, the flies with dark spots in their eyes tended to die earlier than the control flies, and more ommatidial fusions were observed in 20-day-old fly eyes with deposits (marked with asterisks in Fig. 3B3), with further exacerbation of these fusions in 30-day-old fly eyes (marked with asterisks in Fig. 3B4). **(C**–**E)** Acacetin partially suppressed the morphological defects in the eyes. Scanning electron micrographs. **(F1**,**G1)** Thirty-day-old male *GMR-GAL4*/+ flies with smooth surface compound eyes, without any defects in the ommatidia size, ommatidial bristles, or fused ommatidia. **(F2**,**G2)** Vehicle-fed male *GMR* < *APP/BACE-1* flies (30 days old) with abnormal phenotypes, characterized by the collapse (marked with arrows) and fusion of ommatidia (marked with arrowheads), and the absence of ommatidial bristles (marked with an asterisk). **(F3–5**,**G3–5)** Acacetin partially suppressed these eye defects. The scale bars represent 20 μm.

It has been reported that co-expression of the human *APP* and *BACE-1* genes induced the age-dependent neurodegeneration of the photoreceptor cells in *Drosophila* compound eyes^31^. To evaluate whether acacetin affected eye degeneration over time, the morphological changes in the eyes were first investigated in flies of different ages (1, 10, 20, and 30 days old). The control strain carrying *GMR-GAL4* alone showed a normal, well-organized, and smooth external eye surface, and there were no obvious morphological changes as time elapsed (Fig. 3A1–A4). In contrast, external phenotypic changes were observed in flies co-expressing human *APP* and *BACE-1*, and these changes became more severe as time elapsed (Fig. 3B–E). In vehicle-fed flies, dark deposits were observed at the edges of the compound eyes, even in 1-day-old flies (marked with an arrow in Fig. 3B1), and the size of the deposits had increased in 10-day-old flies (marked with an arrow in Fig. 3B2). As time elapsed, the flies with dark spots in their eyes died earlier than those without the dark spots. Furthermore, the number of ommatidial fusions increased in 20-day-old fly eyes compared to the 1-day-old fly eyes (marked with asterisks in Fig. 3B3) and were further exacerbated in 30-day-old fly eyes (marked with asterisks in Fig. 3B4).

Next, *GMR* < *APP/BACE-1* flies in the egg stage were cultured in media supplemented with acacetin (100, 300, and 500 μM) dissolved in 0.1% DMSO. Acacetin suppressed the numbers of flies with dark spots in their eyes (Fig. 2) at 1 day, although there were no obvious external structural changes in acacetin-fed fly eyes at 1 and 10 days old (Fig. 3C1–E1). However, the visible eye color changed from light red to dark red in 20- or 30-day-old flies (Fig. 3C3–E3). Furthermore, the compound eyes in these older flies had a rougher surface and more severe ommatidial fusions than those in the younger flies; this was especially pronounced for the 30-day-old flies (marked with asterisks in Fig. 3C4–E4). At concentrations of 100, 300, and 500 μM, acacetin significantly suppressed ommatidial fusion in the central side of the flies’ eyes compared to the vehicle-fed flies (marked with an asterisk in Fig. 3B3–E3). In particular, 300 and 500 μM acacetin ameliorated ommatidial fusion at the edges of the fly eyes. Although acacetin did not completely suppress the morphological changes in the eyes, it delayed this age-dependent degeneration progress.

The protective effects of acacetin on the compound eyes of the male *GMR* < *APP/BACE-1* flies (30 days old) were examined using SEM. The control *GMR-GAL4*/+ flies had eyes with a smooth appearance, without any defects in the ommatidia size (Fig. 3F1) or ommatidial bristles (Fig. 3G1). In contrast, the transgenic *GMR* *<* *APP/BACE-1* flies showed varying degrees of eye disorganization. The eyes of the vehicle-fed flies showed the strongest phenotypes; they were characterized by ommatidial collapse (marked with arrows in Fig. 3F2), fused ommatidia (marked with arrowheads in Fig. 3G2), a reduced ommatidia size (marked with an asterisk in Fig. 3G2), and the absence of ommatidial bristles (Fig. 3G2). These eye phenotype defects were partially suppressed in male flies treated with three concentrations of acacetin (100, 300, and 500 μM) (Fig. 3F3, 4, 5,G3, 4, 5). Remarkably, external surface collapse was not observed in flies fed 300 μM acacetin (Fig. 3F4), but there was still some collapse in flies fed 100 and 500 μM acacetin (marked with arrows in Fig. 3F3,F5). Ommatidial external surface fusion was not observed in acacetin-fed flies, but the reduced ommatidia size (marked with asterisks in Fig. 3G3) was observed, irrespective of concentration.

### Acacetin has a protective effect on age-dependent neurodegeneration of the eye

**Figure 4:**
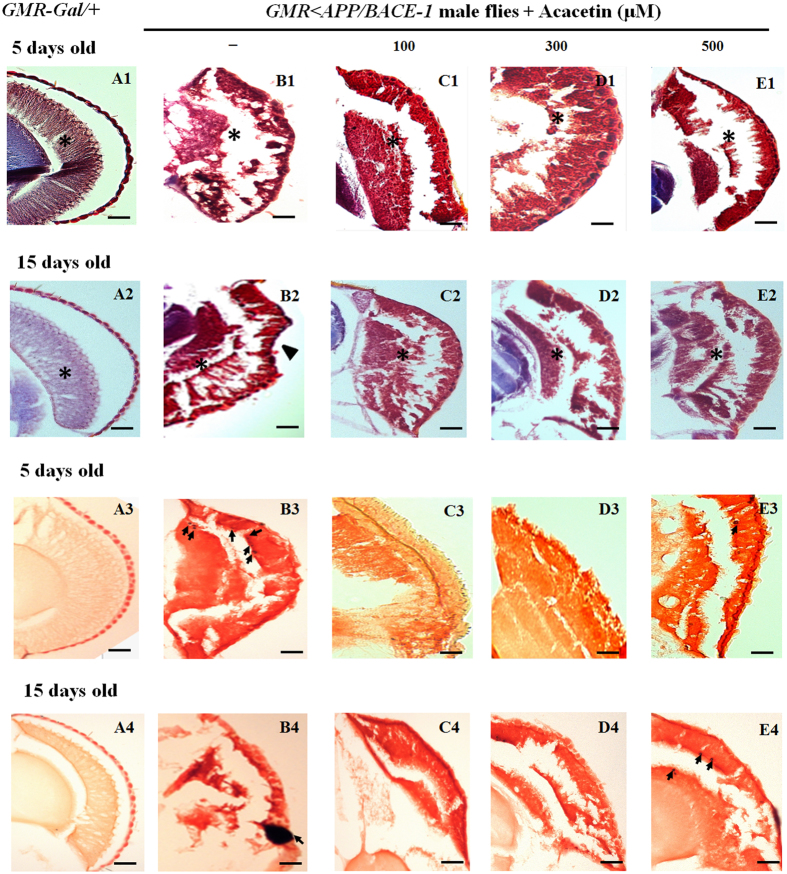
Protective effect of acacetin on age-dependent eye degeneration induced by co-expression of human BACE-1 and APP. Human APP and BACE-1 transgenic flies (*elav* < *APP/BACE-1*) were cultured from the egg stage in polystyrene vials containing standard media supplemented with acacetin (100, 300, and 500 μM) in 0.1% DMSO. Cason’s trichrome staining. (**A1**,**A2)** Staining of the photoreceptors of control male *GMR-Gal4*/+ flies (5 and 15days old). **(B1**,**B2)** Staining of the photoreceptors of vehicle-fed transgenic male *GMR* < *APP/BACE-1* flies (5 and 15days old). Remarkable retinal collapse (marked with an asterisk in B1) was observed even in young flies (5 days old), and more severe external surface collapse of the photoreceptors was detected in 15-day-old flies (marked with an arrowhead in B2) compared to 5-day-old flies. (**C1-E1)** The photoreceptor degeneration was partially suppressed in 5-day-old acacetin-fed transgenic flies. **(C2-E2)** The external surface collapse of the photoreceptors was significantly suppressed in 15-day-old acacetin-fed flies. Congo red staining. **(A3**,**A4)** Amyloid plaques were not observed in the control male *GMR-Gal4*/+ flies (5 and 15 days old). **(B3**,**B4)** Scattered amyloid plaque deposits were apparent in the 5-day-old vehicle-treated transgenic flies (marked with arrows in B3). Many amyloid plaque deposits (marked with an arrowhead in B4) had accumulated in the 15-day-old flies. **(C3-E3)** Acacetin reduced the number of scattered amyloid plaque deposits in the photoreceptors of 5-day-old transgenic flies. **(C4-E4)** Acacetin reduced the number of amyloid plaque deposits in the 15-day-old transgenic flies. The images were observed and captured using a stereo-microscope. The scale bars represent 20 μm.

Male compound eye sections were examined to demonstrate the protective effects of acacetin on the age-dependent neurodegeneration induced by co-expression of human *BACE-1* and *APP*. Photoreceptor degeneration in the transgenic *GMR* < *APP/BACE-1* flies depended on age, but this was not the case for the control *GMR-Gal4*/+ flies (Fig. 4). The *GMR-Gal4*/+ flies (5 and 15 days old) had well-organized photoreceptors, without age-dependent phenomena, as determined by Cason’s trichrome staining (Fig. 4A1). In contrast, photoreceptor degeneration was observed in young transgenic flies (Fig. 4B1–E1) and 15-day-old flies (Fig. 4B2–E2). In the vehicle-fed transgenic flies, remarkable retinal collapse was observed, even in young flies (5 days old) (Fig. 4B1), and external surface collapse of the photoreceptors was detected in the 15-day-old flies (marked with an arrowhead in Fig. 4B2). Acacetin (100, 300, and 500 μM) partially suppressed the photoreceptor degeneration in 5- (Fig. 4C1–E1) and 15-day-old flies (Fig. 4C2–E2). In particular, acacetin significantly suppressed the photoreceptor collapse (Fig. 4C2–E2). Although acacetin did not completely suppress photoreceptor degeneration, it delayed this age-dependent process.

Congo red staining was performed to investigate whether amyloid deposition was age-dependent or due to the effects of acacetin on these deposits. Amyloid plaques were not detected in the compound eyes of the control male *GMR-Gal4*/+ flies (5 and 15 days old) (Fig. 4A3). However, scattered amyloid plaque deposits (marked with arrows in Fig. 4B3) were evident in 5-day-old vehicle-fed transgenic flies, and many amyloid plaque deposits (marked with arrows in Fig. 4B4) had accumulated in the 15-day-old flies. Interestingly, Congo red staining revealed that 100 and 300 μM acacetin significantly suppressed the number of amyloid deposits in 5- (Fig. 4C3) and 15-day-old transgenic flies (Fig. 4C4); however, a few amyloid plaque deposits were also detected in 5- (marked with arrows in Fig. 4E3) and 15-day-old transgenic flies (marked with arrows in Fig. 4E4) supplemented with 500 μM acacetin. Although acacetin did not completely suppress amyloid deposition in the flies’ photoreceptors, it reduced the number of amyloid deposits.

### Effect of acacetin on eclosion of the transgenic flies

**Figure 5:**
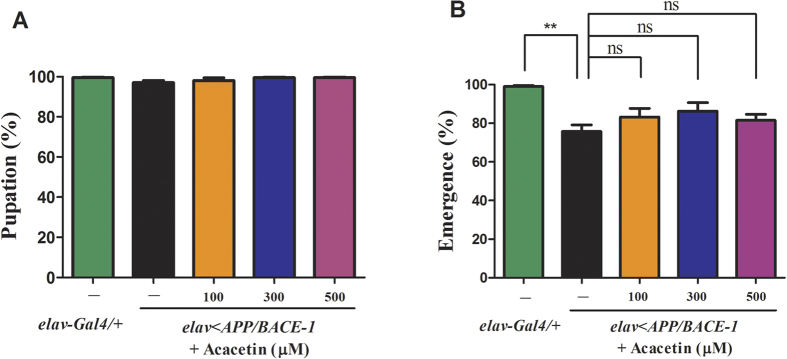
Effect of acacetin on the eclosion rate of the transgenic flies. Human APP and BACE-1 transgenic flies (*elav* < *APP/BACE-1*) were cultured from the egg stage in polystyrene vials containing standard media supplemented with acacetin (100, 300, and 500 μM) in 0.1% DMSO. The third instar larvae climbing on the wall were collected in vials containing fresh media. The numbers of pupae and adults were counted. **(A)** Co-expression of human *BACE-1* and *APP* as along with acacetin supplementation did not affect the pupation of the male *elav-Gal4*/+ and *elav* < *APP/BACE-1* flies. **(B)** Co-expression of human *BACE-1* and *APP* significantly reduced the emergence of the male transgenic flies. Acacetin had no significant effect on the emergence defect. Each bar represents the mean ± SE from four independent experiments (^**^P < 0.01; ns, no significant difference, using Bonferroni’s multiple comparison test).

The pupation and emergence rates were examined to determine whether acacetin affected the development of the transgenic flies. Co-expression of human *BACE-1* and *APP* in the nervous system did not affect pupation compared to the control *elav-Gal4*/+ flies. The acacetin-treated flies showed nearly normal pupation rates, irrespective of the treatment concentration (100, 300, and 500 μM), and these rates were not significantly different from those of the vehicle-fed transgenic flies (Fig. 5A). This could be due to the relatively weak promoter – *elav* – used to drive human *APP* and *BACE-1* gene expression. However, the emergence rate (76%) of the vehicle-fed *elav* < *APP/BACE-1* flies was significantly different from that (99%) of the *elav-Gal4*/+ flies (Fig. 5B). This finding indicates that co-expression of human *BACE-1* and *APP* is toxic during fly development, particularly at the emergence stage. Acacetin (100, 300, and 500 μM) did not have a significant effect on the emergence of flies (82–86%) compared to the vehicle.

### Effect of acacetin on the flies’ age-dependent motor abnormalities

**Figure 6:**
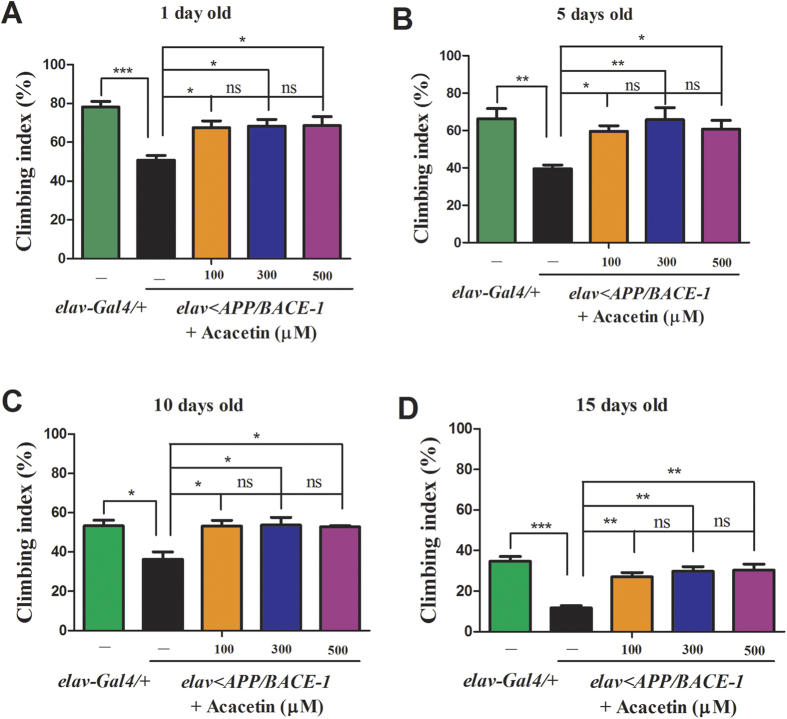
Effect of acacetin on the age-dependent climbing behavior of the transgenic flies. Human APP and BACE-1 transgenic flies (*elav* < *APP/BACE-1*) were cultured from the egg stage in polystyrene vials containing standard media supplemented with acacetin (100, 300, and 500 μM) in 0.1% DMSO. Male flies (1, 5, 10, and 15 days old) were placed in empty vials conjoined with another vial on top and manually tapped twice. After 20 s, the flies that climbed from the bottom and crossed the 9.5 cm line were counted, and the climbing index was calculated as the number of flies that climbed to the top vial relative to the total number of test flies and expressed as a percentage. Climbing behavior of 1- **(A)**, 5- **(B)**, 10- **(C)**, and 15-day-old male flies **(D)**. Co-expression of human *BACE-1* and *APP* significantly decreased the flies’ climbing activities compared to the control *elav-Gal4*/+ flies. Acacetin ameliorated the climbing defects of the *elav* < *APP/BACE-1* flies compared to the vehicle-fed flies, irrespective of concentration. Each bar represents the mean ± SE from three independent experiments (^***^P < 0.001; ^**^P < 0.01; ^*^P < 0.05; ns, no significant difference, using Bonferroni’s multiple comparison test).

AD is characterized by age-dependent degeneration in locomotor coordination^32^. In *Drosophila* AD models, locomotor coordination can be quantified by the negative geotaxis assay, as described by Wang *et al.*^33^ and Crowther and colleagues^34^. We used male flies because the climbing defects were more pronounced in males than in females. The male *elav-Gal4*/+ flies showed a clear age-dependent reduction in climbing, with climbing indices of 80, 66, 53, and 34% at 1, 5, 10, and 15 days postemergence, respectively (Fig. 6A–D). In contrast, the vehicle-fed male *elav* < *APP/BACE-1* flies showed severe defects in performance, with climbing indices of 47, 39, 36, and 11% at 1, 5, 10, and 15 days postemergence, respectively (Fig. 6A–D). Acacetin improved the motor abnormalities to approximately 68% as early as 1 day after emergence, irrespective of the concentration (100, 300, and 500 μM) (Fig. 6A). The acacetin treatment resulted in a 60–66% climbing index at 5 days postemergence (Fig. 6B) and a 53–54% climbing index at 10 days postemergence (Fig. 6C). At 15 days postemergence, acacetin significantly ameliorated the climbing defects (climbing index, 27–30%) (Fig. 6D).

### Effect of acacetin on fly feeding and longevity

**Figure 7:**
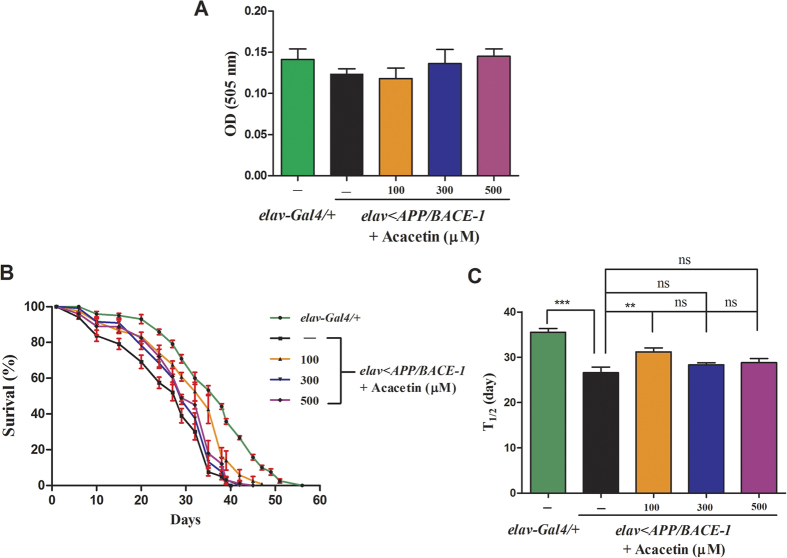
Effect of acacetin on the feeding and longevity of the transgenic flies. Human APP and BACE-1 transgenic flies (*elav* < *APP/BACE-1*) were cultured from the egg stage in polystyrene vials containing standard media supplemented with acacetin (100, 300, and 500 μM) in 0.1% DMSO to determine the lifespan and median life time (T_1/2_). **(A)** To determine feeding behavior, newly emerged transgenic male flies were cultured on standard media for 3 days, and then starved for 20 h. The flies were transferred into vials containing media (with 0.2% Acid red) supplemented with acacetin. The control flies were fed with media containing 0.2% Acid red and vehicle. The abdomens of flies fed with Acid red were cut and homogenized in distilled water, and the optical density (OD) of the supernatant was measured at 505 nm as the index of amount of food consumed by the flies. Acacetin did not affect the feeding behavior of the flies. **(B)** Acacetin did not significantly prolong the transgenic flies’ lifespan compared to the vehicle-fed male transgenic and *elav-Gal4*/+ flies. **(C)** Co-expression of human *BACE-1* and *APP* reduced the T_1/2_ of the male flies compared to that of the control male *elav-Gal4*/+ flies. Supplementing the diet with 100 μM acacetin only significantly extended the T_1/2_ of the male *elav* < *APP/BACE-1* flies. Each bar represents the mean ± SE from 10 independent experiments for longevity and from three independent experiments for feeding (^***^P < 0.001; ^**^P < 0.01; ns, no significant difference, using Bonferroni’s multiple comparison test).

A feeding assay was used as an index to quantify the effect of different concentrations of the active compound on the amount of food consumed by the flies^35^,^36^. The effects of acacetin on food intake were examined in transgenic male *elav* < *APP/BACE-1* flies. Acacetin did not significantly affect feeding by transgenic flies, irrespective of the concentration (100, 300, and 500 μM) (Fig. 7A), indicating that any changes in the flies’ behaviors were due to the ingestion of compounds by food intake rather than the effect of acacetin on their appetite.

The median life time (T_1/2_) is a more credible measurement of lifespan than the mean survival time^37^. We investigated the effects of acacetin on the lifespan and T_1/2_ of our *Drosophila* AD model, because it was previously reported that co-expression of *BACE-1* and *APP* reduced the lifespan of the adult flies^37^. Acacetin (100, 300, and 500 μM) did not significantly increase the lifespan of the male *elav* < *APP/BACE-1* flies compared to the vehicle-fed transgenic flies and control *elav-Gal4*/+ flies (Fig. 7B). However, co-expression of human *BACE-1* and *APP* markedly reduced the T_1/2_ (26 days) of vehicle-fed flies compared to the *elav-Gal4*/+ flies (T_1/2_, 36 days) (Fig. 7C). Treatment of transgenic flies with 100 μM acacetin increased the T_1/2_ (31 days), while 300 and 500 μM acacetin did not significantly extend the T_1/2_. These findings suggest that the lowest concentration of acacetin (100 μM) has an effect on the T_1/2_ of *elav* < *APP/BACE-1* flies; however, this is limited to young adults. Taken together, these data suggest that BACE-1 may not have a major function in the survival of *elav* < *APP/BACE-1* flies, which is consistent with the conclusions of a previous study^37^.

### Effect of acacetin on the human *APP* and *BACE-1* mRNA levels

**Figure 8:**
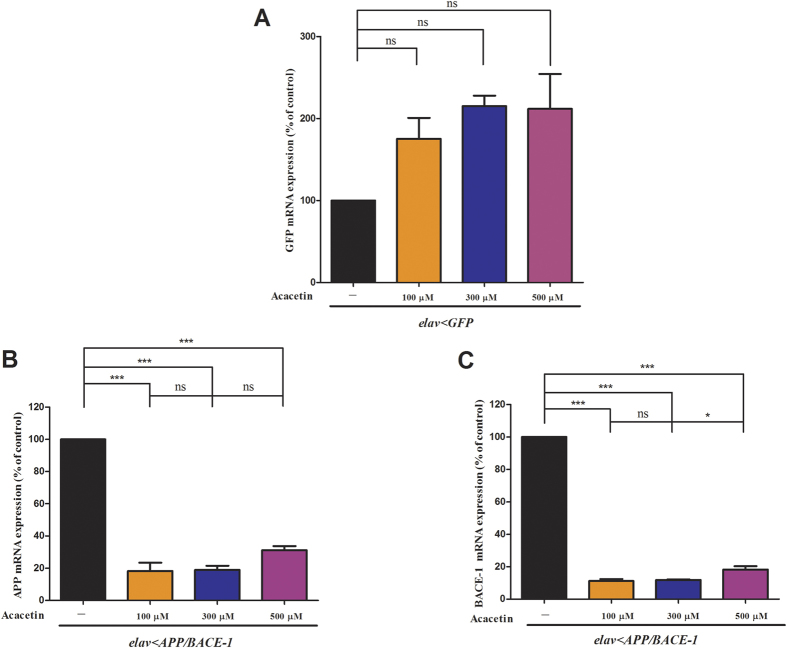
Effect of acacetin on the expression of the human BACE-1 and APP mRNAs. The total RNA was extracted from 30 heads (30 mg) of *elav* < *APP/BACE-1* and *elav* < *GFP* flies (20 days old) cultured from the egg stage in polystyrene vials containing media supplemented with acacetin (100, 300, and 500 μM) in 0.1% DMSO. Real-time qRT-PCR was performed to determine the levels of the *BACE-1* and *APP* mRNAs. Specific *BACE-1*, *APP*, and ribosomal protein 49 (*rp 49*) coding sequence primers were used to amplify the *BACE-1*, *APP*, and *rp 49* cDNAs, as described in the Methods section. **(A)** The *elav* < *GFP* flies were used as control flies to confirm that the effects of acacetin to reduce the APP and BACE-1 levels were not due to its inhibitory activity toward the Gal4 transcription activator. Acacetin had no significant effects on *GFP* mRNA expression, irrespective of concentration. **(B)** Acacetin significantly reduced the human *APP* mRNA levels. **(C)** Acacetin significantly reduced human *BACE-1* mRNA expression in 20-day-old male flies. The mRNA expression was normalized to the constitutive expression of the mRNA for the housekeeping gene, *rp 49*, and analyzed by the 2^–ΔΔ*C*T^ method. Each bar represents the mean ± SE of duplicate samples run in three independent experiments (^***^P < 0.001; ^*^P < 0.05; ns, no significant difference, using Bonferroni’s multiple comparison test).

Many studies have focused on APP proteolysis and Aβ generation as potential targets for AD therapy^38^, and APP inhibitors are also used to lower the Aβ peptide levels, as described by Utsuki *et al.*^39^. To investigate whether acacetin affected the transcription of the human *BACE-1* and *APP* genes, we analyzed the *APP* and *BACE-1* mRNA levels in male *elav* < *APP/BACE-1* flies (20 days old) using real-time qRT-PCR. An active compound could reduce the human *APP* and *BACE-1* mRNA levels by indirectly inhibiting the *elav* promoter. To investigate this, we used flies with a *UAS-GFP* sequence driven by the *elav* promoter and treated them with acacetin. We also quantified the levels of the *GFP* mRNA. There were no significant differences in the *GFP* mRNA levels between the vehicle-fed and acacetin-fed flies (Fig. 8A). However, a significant reduction in the *APP* mRNA levels (69–82%) was observed in the male *elav* < *APP/BACE-1* flies following treatment with acacetin (100, 300, and 500 μM) compared to the vehicle-treated flies (Fig. 8B). Similarly, the human *BACE-1* mRNA levels were reduced to 82–89% of the control levels following acacetin treatment (Fig. 8C). Taken together, these data indicate that acacetin had no significant effect on the *GFP* mRNA levels, but decreased the human *APP* and *BACE-1* mRNA levels rather than indirectly inhibiting the *elav* promoter.

### Acacetin significantly reduces the Aβ levels by interfering with human APP proteolytic processing and BACE-1 expression

**Figure 9:**
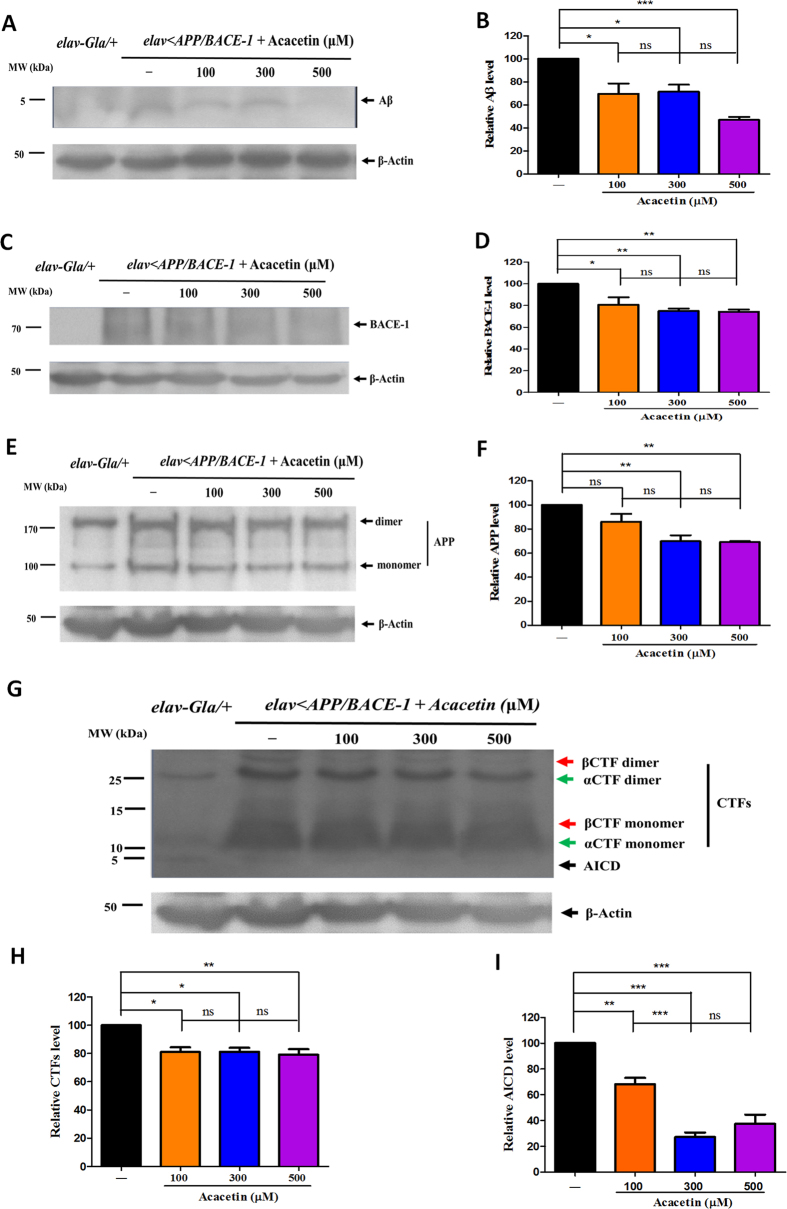
Effect of acacetin on Aβ, BACE-1, and APP processing in the transgenic flies. Human APP and BACE-1 transgenic flies (*elav* < *APP/BACE-1*) were cultured from the egg stage in polystyrene vials containing media supplemented with acacetin (100, 300, and 500 μM) in 0.1% DMSO. Western blot analyses were performed to determine the levels of the Aβ, BACE-1, APP, APP-CTFs, and AICD proteins, as described in the Methods section. **(A)** Aβ was immunoblotted with an Adβ, 17–24 (4G8) monoclonal antibody, and each lane contained 70 μg of protein. **(B)** The relative amounts of total Aβ were detected by western blotting. **(C)** BACE-1 was probed with an anti-BACE-1 antibody, and each lane contained 30 μg of protein. **(D)** Protein levels of human BACE-1 in the transgenic flies. **(E)** Human APP was detected by western blotting with an anti-APP C-terminal antibody. Each lane contained 30 μg of protein. **(F)** Quantification of the human APP protein levels in the transgenic flies. **(G)** The APP proteolytic processing products βCTF, αCTF, and AICD were detected by western blotting with an anti-APP C-terminal antibody; each lane contained 30 μg of protein. **(H)** The levels of the CTFs resulting from APP processing in the transgenic flies. **(I)** The levels of the AICD fragment resulting from APP processing in the transgenic flies. β-Actin was used as a loading control. The differences in protein expression were quantified using a Molecular Imager Gel Doc XR system and normalized to the actin expression on the same membrane. Each bar represents the mean ± SE of duplicate samples of three independent experiments (^***^P < 0.001; ^**^P < 0.01; ^*^P < 0.05; ns, no significant difference, using Bonferroni’s multiple comparison test).

A western blot analysis revealed that acacetin suppressed Aβ expression, although the responses varied according to concentration. Aβ (approximately 4 kDa) was not detected in the control *elav-Gal4*/+ flies (Fig. 9A). Quantification of the western blots showed that 100, 300, and 500 μM acacetin decreased the Aβ levels to 71, 72, and 47% of the controls, respectively (Fig. 9B). To determine whether the anti-amyloidogenic effect of acacetin was mediated by modulating APP proteolytic processing or BACE-1 expression, the protein levels of BACE-1 and APP were analyzed. A band corresponding to BACE-1 (approximately 70 kDa) was visible in the transgenic flies, but was not detected in the control *elav-Gal4*/+ flies. Treatment with 100, 300, and 500 μM acacetin decreased the expression of BACE-1 (Fig. 9C). Quantification of the western blots indicated that the BACE-1 protein levels were considerably decreased to 81, 75, and 74% of the control levels using 100, 300, and 500 μM acacetin, respectively (Fig. 9D). In this experiment, the APP monomer (approximately 100 kDa) and dimer bands (approximately 200 kDa) were confirmed by the western blots, as described by Jung *et al.*^40^, and were also visible in the control *elav-Gal4*/+ flies, which is consistent with the previous study by Groth *et al.*^41^ (Fig. 9E). Quantification of the western blots indicated that the APP levels were also significantly reduced to 70 and 69% of the control levels by 300 and 500 μM acacetin, respectively, but 100 μM acacetin reduced the APP levels to 86%, which was not significantly different from the control APP levels (Fig. 9F). We also analyzed the effects of acacetin on the formation of the APP-alpha C-terminal fragment (αCTF) (14 kDa, marked with the green arrow in Fig. 9G) and APP-βCTF (14.5 kDa, marked with the red arrow in Fig. 9G), because the level of the APP-CTF is useful for understanding how genetic manipulation of APP processing impacts Aβ generation and accumulation. Acacetin inhibited the generation of the APP-CTF by affecting APP cleavage (approximately 80% of the control levels) (Fig. 9H). The APP intracellular domain (AICD) fragment has been shown to be involved in a variety of signaling processing, many of which are potentially relevant to AD pathology, and the AICD levels are elevated in human AD brains, as described by Branca *et al.*^42^. Accordingly, we also investigated the effects of acacetin on the AICD levels. A single band was detected in both the control *elav-Gal4*/+ and transgenic *elav* < *APP/BACE-1* flies, and acacetin decreased the AICD levels (approximately 7 kDa) in the transgenic flies (Fig. 9G). Quantification of the western blots revealed that the AICD levels were reduced to 68, 27, and 37% of the control levels following treatment with 100, 300, and 500 μM acacetin, respectively (Fig. 9I).

## Discussion

The amyloid cascade hypothesis, which presumes that the deposition of amyloid plaques in the brain is the reason for the AD pathology, has promoted the development of drugs to treat AD^5^,^43^. There are three proteases involved in APP processing to produce amyloid plaques – α-secretase, β-secretase, and γ-secretase—and β-secretase is a major therapeutic target in AD^44^. Selective phytochemicals may be used to treat AD, and are of great interest because they may biodegrade to nontoxic products^45^. These anti-AD products can be applied to humans in the same manner as conventional drugs. Plants contain various compounds, such as alkaloids, phenols, and terpenoids, and these compounds, alone or in combination, contribute to BACE-1 inhibition^14^,^15^. The phytochemicals that inhibit human BACE-1 include alkaloids (e.g., epiberberi and groenlandicine, IC_50_ 8.55 and 19.68 μM, respectively^46^), terpenoids (e.g., 16α-hydroxy-17-isovaleroyloxy-*ent*-kauran-19-oic acid and 10 other diterpenoids, IC_50_ 18.58–92.20 μM^47^; bakuchiol, IC_50_ 21.38 μM^48^), flavonoids (e.g., epigallocatechin gallate and other two catechins, IC_50_ 1.6–4.5 μM^24^; neocorylin and other five flavonoids, IC_50_ 0.7–10.2 μM^48^; kuraridin and other two chalcones, IC_50_ 6.03–7.19^49^; leachianone G and six other flavonones, IC_50_ 8.56–60.88 μM^49^), benzopyranoids (e.g., aloeresin D and C-2′-decoumaroyl-aloeresin G, IC_50_ 39.0 and 20.5 μM, respectively^50^; imperatorin and its four derivatives, IC_50_ 91.8–359.2 μM^51^), phenylpropanoids (e.g., *p*-coumaric acid, IC_50_ 90 μM^52^), stilbenoids (e.g., resveratrol and its eight derivatives, IC_50_ 0.34–19.80 μM^53^), diarylalkyls (e.g., bisdemethoxycurcumin and its two derivatives, IC_50_ 17–340 μM^33^), and tannins (e.g., geraniin and corilagin, IC_50_ 4 and 34 μM, respectively^54^).

In the current study, we used a FRET-based enzyme assay to identify the BACE-1 inhibitory constituents from *A. rugosa* whole plant extracts. The active constituents were determined to be the *O*-methylated flavone acacetin, and the oleanane triterpenoids maslinic acid and oleanolic acid; the chemical structure of oleanolic acid differs from that of maslinic acid by the lack of a hydroxyl group at the 2-carbon position. The IC_50_ values of these constituents were between 88.5 and 487.6 μM, while the IC_50_ values of the natural compounds described above are between 0.34 and 359.2 μM. Acacetin was more potent in inhibiting BACE-1 than either maslinic acid or oleanolic acid, although it was less potent in inhibiting BACE-1 than the human BACE-1 inhibitor IV. However, the peptide-based BACE-1 inhibitor IV and other BACE-1 inhibitors are poorly absorbed when administered orally, due to their low penetrability across the blood brain barrier, which results in little pharmacological activity *in vivo*^23^. Small molecule BACE-1 inhibitors with a low molecular weight and good plasma membrane permeability are crucial for drug development. Many *in vivo* studies have shown that flavonoids can be absorbed by oral administration, cross the blood brain barrier, and work on the central nervous system^55^,^56^. Acacetin has been reported to possess antioxidant^57^, anti-inflammatory^58^, and anticarcinogenic^59^ activities and have neuroprotective effects on the central nervous system. They also have therapeutic potential for treating neurological diseases associated with excitotoxicity^60^.

*Drosophila* models of amyloid toxicity and Tau have been developed to simulate the underlying pathogenesis of AD^61^. Several researchers have evaluated whether phytochemicals have therapeutic effects in *Drosophila* AD models. Caesar *et al.*^62^ studied the behavior of curcumin as a drug candidate to alleviate Aβ toxicity in five different transgenic *Drosophila* AD models. They reported that curcumin treatment resulted in an improved lifespan (up to 75%) and climbing activity in transgenic flies; Aβ deposition was not decreased following treatment. It has also been reported that prolonged exposure to either curcumin or bisdemethoxycurcumin can rescue the morphological defects (ommatidia atrophy at the edges of the compound eye, absence of ommatidial bristles, and ommatidial fusion) in the compound eyes of *GMR* < *APP/BACE-1* flies co-expressing *APP* and *BACE-1* and also significantly improve locomotor coordination in the *elav* < *BACE-1* and *elav* < *APP/BACE-1* flies^33^.

In the current study, the human *APP* and *BACE-1* genes were co-expressed under the control of the *elav* and *gmr* promoters. Some dark spots were observed in the compound eyes of the *GMR* < *APP/BACE-1* flies. Chakraborty *et al.*^37^ reported the presence of melanotic masses on both the ventral abdomen and proboscis of *elav* < *APP/BACE* flies. The number of masses was significantly decreased in these flies after treatment with the γ-secretase inhibitor L-685,458. They suggested that the masses were composed of Aβ or induced by Aβ. A previous study showed that β-amyloid deposits were localized outside the retinas of the *GMR* < *APP/BACE-1* flies^31^. According to these studies, the dark spots in the *Drosophila* eyes might be composed of Aβ due to the expression of the human *APP* and *BACE-1* genes in the photoreceptor cells and secretion of Aβ by these photoreceptor cells into the projection areas^31^. Here, Aβ could induce the immune response by activating the Toll pathway^63^,^64^, resulting in the formation of dark spots on the surfaces of the compound eyes. We found that acacetin could rescue the morphological defects (dark deposition, photoreceptor collapse and fusion, and absence of ommatidial bristles) in the eyes of the *GMR* < *APP/BACE-1* flies that co-express *BACE-1* and *APP* in their compound eyes. In addition, acacetin also improved the locomotor coordination in the *elav* < *APP/BACE-1* flies and prolonged the T_1/2_ of the transgenic flies, without any effects on feeding behavior. These findings, together with our elucidation of the inhibitory action of acacetin on BACE-1, indicate that *A. rugosa* whole plant-derived materials hold promise for the development of novel, effective, naturally occurring anti-AD agents.

An investigation of the mechanisms of action of naturally occurring anti-AD compounds may provide useful information for the development of selective anti-AD therapeutic alternatives with novel target sites^62^. The target sites and mechanisms underlying the anti-dementia actions of plant secondary substances have been well documented by Howes and Perry^65^. In the amyloidogenic pathway, APP is cleaved by BACE-1 and releases a large soluble ectodomain of APP and APP-βCTF, which is then further cleaved by γ-secretase to generate toxic Aβ^13^,^66^. The protein levels and enzymatic activity of BACE-1 are elevated in AD brains, suggesting that abnormal BACE-1 regulation may significantly contribute to AD pathogenesis^67^. Utsuki *et al.*^39^ screened 144 analogs of phenserine, a physostigmine analog, to identify small molecules that inhibit APP protein synthesis and the subsequent Aβ production, without possessing potent AChE inhibitory activity, using an enzyme-linked immunosorbent assay. They reported eight analogs, including posiphen, an (–)-enantiomer of phenserine, that were capable of dose-dependently reducing APP and Aβ production without causing cell toxicity. These analogs also inhibited APP synthesis, resulting in a decrease in the number of amyloid plaques. Natural flavonoids, such as myricetin and quercetin, have also been reported to be potent inhibitors of BACE-1 activity and to reduce the Aβ levels in primary cortical neurons^68^. Long-term treatment with the EGb761 *Ginkgo biloba* extract significantly lowered the APP protein levels in a transgenic AD mouse model, suggesting that the potential neuroprotective properties of EGb761 may be, at least in part, related to its APP lowering activity^69^. In addition, certain flavonoids and their metabolites have been shown to exert beneficial effects on neurological processes through their interaction with neuronal signaling pathways, such as the PI3K/Akt, tyrosine kinase, protein kinase C, and MAPK signing pathways, as well as the nuclear factor-κB pathway. Inhibitory or stimulatory effects on these pathways are likely to have a large impact on neuronal function by modulating gene expression^7^.

In the current study, the real-time qRT-PCR analysis revealed that acacetin was able to reduce both the human *APP* and *BACE-1* mRNA levels in a transgenic *Drosophila* AD model, without significantly affecting the *GFP* mRNA levels. This finding suggests that acacetin plays an important role in inhibiting human BACE-1 and APP by regulating gene transcription, and it does not block binding of the transcriptional activator Gal4 to the UAS activation domain. Western blot analysis revealed that acacetin reduced Aβ production by interfering with BACE-1 activity and APP synthesis, resulting in a decrease in the levels of APP-CTF and AICD. Therefore, the protective effect of acacetin on Aβ production is mediated by transcriptional regulation of the *BACE-1* and *APP* genes, which results in decreased APP levels and BACE-1 activity. Acacetin acts as a direct inhibitor of BACE-1 activity and regulates the expression of both APP and BACE-1. Previous studies^49^,^68^ demonstrated that flavonoids exhibited inhibitory activity against BACE-1 in cell-free and cell-based systems. The flavonoids epicatechin and epigallocatechin were reported to be potent inhibitors of APP processing^70^. It has also been reported that the flavonoid icariin possesses the ability to decrease amyloid deposition in a transgenic mouse model by reducing APP and BACE-1 expression^71^. Acacetin was reported to exert its anti-inflammatory activity by downregulating pro-inflammatory mediators via inhibition of the NF-κB signaling pathways^72^. Furthermore, acacetin enhanced the phosphorylation of p38 mitogen-activated protein kinase (p38 MAPK), which activates the classical Ras/MAPK pathway, to induce neuritogenesis and neuronal differentiation^73^; therefore, acacetin is a potential therapeutic agent for AD.

In conclusion, *A. rugosa* whole plant constituents, particularly acacetin, are potential therapeutics or lead compounds for the prevention or treatment of AD. The anti-AD action of acacetin provides an indication of at least one of the pharmacological actions of *A. rugosa*. Further research is needed on the practical applications of plant-derived preparations as novel anti-AD products to establish their safety profiles in humans and determine whether the activity measured in flies would also occur *in vivo* in humans after consumption of *A. rugosa* whole plant-derived products. *A. rugosa* is used as a wild vegetable and herbal drug in traditional therapies^22^. In addition, detailed tests are needed to understand how to improve the anti-AD potency and stability of the compounds isolated from *A. rugosa* for eventual commercial development.

## Methods

### Experimental groups

**Figure 10:**
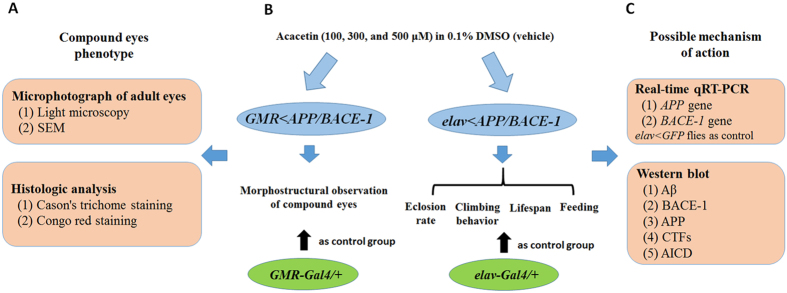
Schematic of the experimental fly groups used in this study. The flies used in this study were cultured from the egg stage in 94 × 25 mm polystyrene vials containing standard media supplemented with acacetin (100, 300, and 500 μM) in DMSO, with the exception of the feeding assay. Newly emerged male flies were cultured on standard media supplemented with acacetin for the feeding assay. **(A)** *GMR-Gal4* drives the co-expression of the human *BACE-1* and *APP* genes in the flies’ compound eyes, and the morphological changes in the compound eyes of these flies were tested. The *GMR-Gal4*/+ flies were used as the control group. (**B)** The *elav-Gal4* promotor drives the co-expression of the targeted transgenes in the flies’ nervous system, and the behavior (climbing and feeding), eclosion rate, and lifespan of the *elav* *<* *APP/BACE-1* flies were tested. The *elav-Gla4*/+ flies were used as the control group. **(C)** The possible mechanism of the anti-AD action of acacetin was elucidated using qRT-PCR and western blot analyses.

In our experiment, acacetin, maslinic acid, and oleanolic acid isolated from the whole *A. rugosa* plant were tested *in vitro* using a FRET-based enzyme assay. Among these constituents, acacetin was tested in flies because it was a more potent inhibitor of BACE-1 than oleanolic acid or maslinic acid. The experimental groups examined for the *in vivo* study are illustrated in Fig. 10. The flies used in this study were cultured from the egg stage in 94 × 25 mm polystyrene *Drosophila* vials (Hansol Tech, Seoul, Republic of Korea (ROK)) containing standard media supplemented with acacetin in 0.1% DMSO, with the exception of the feeding assay. Based on the preliminary test results, the behavior and eye morphology of *Drosophila* models were determined with three concentrations (100, 300, and 500 μM) of acacetin. Newly emerged male flies were cultured on standard media supplemented with acacetin for the feeding assays. *GMR-Gal4* drove the co-expression of human *APP* and *BACE-1* in the compound eyes of the flies, while *GMR-Gal*/+ flies were used as the control group. The age-dependent morphological changes in the flies’ compound eyes were observed using light microscopy and SEM. The compound eyes’ phenotypes were also observed by histologic analysis; Cason’s trichrome staining was used to distinguish the histologic changes, while Congo red staining was used to detect the amyloid plaques in the compound eyes (Fig. 10A). The *elav-Gal4* promoter drove the co-expression of the targeted transgenes in the nervous system; *elav-Gal4*/+ was used as the control group. The behavior (climbing and feeding), eclosion (pupation and emergence) rate, and lifespan of the flies were also tested (Fig. 10B). In the Gal4 system, the promoter (or enhancer) drives the expression of the yeast transcriptional activator Gal4 in cell- and tissue-specific patterns, and Gal4, in turn, directs the transcription of Gal4-UAS target genes in an identical pattern^74^. To confirm that the effects of acacetin on human *APP* and *BACE-1* mRNA expression were not due to a promoter (or enhancer) effect in the Gal4 system, the levels of the *GFP* mRNA were compared in vehicle-fed and acacetin-fed male *elav* < *GFP* flies by qRT-PCR. The possible mechanisms of the anti-AD action of acacetin were elucidated using western blotting analyses (Fig. 10C).

### Instrumental analysis

The ^1^H and ^13^C NMR spectra were recorded in MeOD or DMSO-*d*_6_ on an AVANCE 600 spectrometer (Bruker, Rheinspettem, Germany) at 600 and 150 MHz, respectively, using tetramethylsilane as an internal standard. The chemical shifts are given in δ (ppm). The UV spectra were obtained in methanol on a UVICON 933/934 spectrophotometer (Kontron, Milan, Italy), the mass spectra on a JMS-DX 303 spectrometer (Jeol, Tokyo, Japan), and the FT-IR spectra on a Nicolet Magna 550 series II spectrometer (Midac, Irvine, CA, USA). Silica gel 60 (0.063–0.2 mm) (Merck, Darmstadt, Germany) was used for column chromatography. Merck precoated silica gel plates (Kieselgel 60 F_254_) were used for analytical thin layer chromatography (TLC). An Isolera One medium-pressure liquid chromatograph (Biotage, Uppsala, Sweden) and an Agilent 1200 series high-performance liquid chromatograph (Agilent, Santa Clara, CA, USA) were used to isolate the active compounds.

### Materials

Commercially available pure organic acacetin and bovine serum albumin (BSA) were purchased from Santa Cruz Biotechnology (Dallas, TX, USA). BACE-1 inhibitor IV and Acid red were supplied by Merck (Darmstadt, Germany) and Amresco (Cochran Road Solon, OH, USA), respectively. RIPA buffer and the mammalian cell protease inhibitor cocktail were purchased from Sigma-Aldrich (St. Louis, MO, USA). Recombinant human BACE-1 and the fluorogenic peptide substrate Mca-SEVNLDAEFRK (Dnp) RR-NH_2_ were purchased from R&D Systems (Minneapolis, MN, USA). The PageRuler Prestained Protein Ladder and Spectra Multicolor Low Range Protein Ladder were purchased from Thermo Scientific (Walldorf, Germany). All of the other chemicals and reagents used in this study were of reagent-grade quality and are available commercially.

### Antibodies

The primary antibodies used in this study were as follows: APP C-terminal antibody (A8717) from Sigma-Aldrich; anti-BACE-1 antibody (ab2077) and anti-actin antibody (ab1801) from Abcam (Cambridge, MA, USA); and Aβ, 17–24 (4G8) monoclonal antibody (SIG-39240) from Covance (Princeton, NJ, USA). The secondary antibodies used in this study were goat anti-rabbit IgG H&L (HRP) (ab6721–1) from Abcam and goat anti-mouse IgG-HRP (sc-2005) from Santa Cruz Biotechnology.

### Plant material

Whole *A. rugosa* plants were purchased from the Boeun medicinal herb shop (Seoul Yangnyeongsi, Seoul, ROK). A voucher specimen (AR-WP-01) was deposited in the Research Institute of Agriculture and Life Sciences at Seoul National University.

### *Drosophila* stocks and rearing conditions

The flies were cultured on standard cornmeal agar medium^75^ at 29 °C and 60% relative humidity under a 12:12 h light:dark cycle. The *w1118* (stock number, 3605), *UAS-APP/BACE-1* (33797), *elav-GAL4* (8760), and *GMR-GAL4* (1104) fly stocks used in this study were purchased from the Bloomington *Drosophila* Stock Center at Indiana University and the *UAS-mCD8-GFP/cyo* fly stock was obtained from Dr. Young Ho Koh, Ilsong Institute of Life Science, Hallym University (Anyang, Gyeonggi, ROK). The GAL4/UAS system was used to overexpress the target genes in specific tissues. The characterization of the human APP and BACE-1 transgenic flies (*elav* < *APP/BACE-1* and *GMR* < *APP/BACE-1*) as reliable AD models is described in our previous study^33^.

### Bioassay-guided fractionation and isolation

Air-dried whole *A. rugosa* plants (5 kg) were pulverized, extracted with methanol (3 × 15 L) at room temperature for 2 days, and filtered. The combined filtrate was concentrated to dryness by rotary evaporation at 40 °C to yield approximately 455 g of a black tar. The extract (400 g) was sequentially partitioned into hexane- (75.4 g), chloroform- (55.1 g), ethyl acetate- (27.6 g), butanol- (54.5 g), and water-soluble (187.4 g) portions for the subsequent bioassays. The organic solvent-soluble portions were concentrated under vacuum at 40 °C, and the water-soluble portion was concentrated at 50 °C. To isolate the active constituents, 0.1–2 mg/mL of each *A. rugosa* whole plant-derived fraction was tested in a FRET-based enzyme assay, as described by Wang *et al.*^33^ and Lv *et al.*^50^.

**Figure 11:**
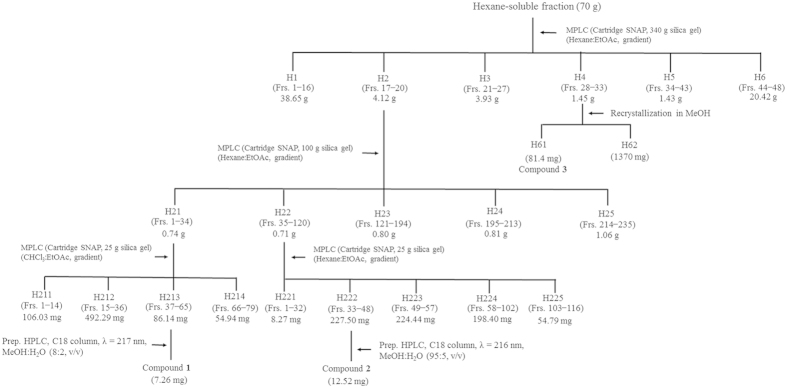
Procedures to isolate the BACE-1 inhibitory constituents. The *Agastache rugosa* whole plant methanol extract was sequentially partitioned into hexane-, chloroform-, ethyl acetate-, butanol-, and water-soluble portions. The hexane-soluble fraction was the most biologically active fraction, and MPLC was performed. Each fraction (0.1–2 mg/L) was tested in a FRET-based enzyme assay to isolate the active compounds from the fraction.

The hexane-soluble fraction (10 g) was the most biologically active fraction (Table 1), and medium-pressure liquid chromatography (MPLC) was performed using an Isolera apparatus equipped with a UV detector at 254 and 365 nm and a SNAP column cartridge (340 g silica gel) with a column volume of 510 mL (Fig. 11). Separation was achieved with a gradient of hexane and ethyl acetate (100:0, 90:10, 80:20, 70:30, 40:60, 50:50, 30:70, and 10:90 by volume) and finally with methanol (1 L) at a flow rate of 50 mL/min to provide 48 fractions (each approximately 180 mL). The column fractions were monitored by TLC on silica gel plates developed with a hexane and ethyl acetate (6:4 by volume) mobile phase. Fractions with similar *R*_f_ values on the TLC plates were pooled and the spots were detected by spraying the plate with 2% sulfuric acid. This separation procedure was repeated seven times. Active fractions 17–20 (H2) and 28–33 (H4) were obtained. Fraction H2 was separated by MPLC with a UV detector and a column cartridge (100 g silica gel) with a column volume of 132 mL by elution with a gradient of hexane and ethyl acetate (100:0, 90:10, 80:20, 70:30, 60:40, 50:50, and 30:70 by volume) and finally with 500 mL methanol at a flow rate of 30 mL/min to provide 235 fractions (each approximately 22 mL). The column fractions were monitored by TLC on silica gel plates, as stated previously. Active fractions 1–34 (H21) and 35–120 (H22) were obtained. Fraction H21 was separated by MPLC with a UV detector and a column cartridge (25 g silica gel) with a column volume of 33 mL by elution with a gradient of chloroform and ethyl acetate (100:0, 95:5, 90:10, 85:15, 80:20, 70:30, and 50:50 by volume) and finally with 300 mL methanol at a flow rate of 25 mL/min to provide 79 fractions (each approximately 22 mL). Fraction H22 was separated by MPLC with a UV detector and a column cartridge (25 g silica gel) with a column volume of 33 mL by elution with a gradient of hexane and ethyl acetate (100:0, 95:5, 90:10, 85:15, 80:20, 70:30, 60: 40, 40:60, and 30:70 by volume) and finally with 300 mL methanol at a flow rate of 25 mL/min to give 116 fractions (each approximately 22 mL). A preparative high-performance liquid chromatograph was used to separate the constituents from active fractions 37–65 (H213) from H21 and fractions 33–48 (H222) from H22. The column was a 7.8 mm i.d. × 300 mm μBondapak C18 (Waters, Milford, MA, USA) with a mobile phase of methanol and water (80:20 and 95:5 by volume) at a flow rate of 1 mL/min. Chromatographic separation was monitored using a UV detector at 217 and 216 nm, respectively. Finally, two active constituents, **1** (7.26 mg) from H213 and **2** (12.52 mg) from H222, were isolated at retention times of 13.15 and 16.86 min, respectively. Fraction H4 was recrystallized in methanol at –20 °C to afford active constituent **3** (81.4 mg).

### FRET-based enzyme assay

The methods of Wang *et al.*^33^ and Lv *et al.*^50^ were used, with slight modifications, to assess the BACE-1 inhibitory activity of the test compounds. Briefly, assay mixtures containing 1 μL of 0.5 μg/μL recombinant human BACE-1, 0.75 μL of a 2.5 μg/μL fluorogenic peptide substrate, 47.25 μL of 50 mM sodium acetate (pH 4.5), and the isolated compounds (1–1000 μg/mL) in 2% DMSO were preincubated for 1 h at 25 °C in darkness, followed by the addition of 16.6 μL of 2.5 M sodium acetate to terminate the reaction. The fluorescence intensity was measured at room temperature using a SpectraMAX Gemini XS plate reader (Molecular Devices, Sunnyvale, CA, USA) at 355 nm excitation and 405 nm emission. The inhibition percentage was determined using the following equation: % inhibition = 100−[(*F*_*S*_ − *F*_*S0*_)/(*F*_*C*_ − *F*_*C0*_)] × 100, where *F*_*S*_ and *F*_*S0*_ are the fluorescence of the samples at 60 min and 0 min, and *F*_*C*_ and *F*_*C0*_ are the fluorescence of the control at 60 min and 0 min, respectively^50^. The results are expressed as the means ± standard errors (SEs) of triplicate samples from three independent experiments.

### Light and scanning electron microscopy of the adult eyes

*GMR* < *APP/BACE-1* flies were cultured from the egg stage in polystyrene vials containing standard media supplemented with acacetin (100, 300, and 500 μM) in 0.1% DMSO, based on the preliminary test results. The controls received 0.1% DMSO only. The male flies (1, 10, 20, and 30 days old) were anesthetized on ice and placed on a microscope slide at room temperature for light microscopy. The morphology of the compound eyes was observed using an EZ4 HD stereo microscope (×35) equipped with an Integrated 3.0 Mega-Pixel CMOS camera (Leica, Hicksville, NY, USA). The flies with aberrant eye phenotypes were collected, and the number of the flies with dark deposits in the eye was counted.

For SEM, the anesthetized male flies (30 days old) were attached to a copper mount using silver paint as a conducting adhesive, as described previously^31^. They were then put directly into the viewing chamber of an SEM without prior coating, as described by Hartman and Hayes^76^. The external surface morphology of the compound eye was visualized using a Supra 55VP field-emission scanning electron microscope (Carl Zeiss, Jena, Germany) operated at 15 kV.

### Histologic analysis

Cason’s trichrome staining was performed, as described by Wang *et al.*^33^ and Perumalsamy *et al.*^77^. Briefly, the heads of 5- and 15-day-old male *GMR* < *APP/BACE-1* flies, cultured in vials as stated previously, were fixed in a 4% paraformaldehyde buffer solution (pH 7.4) overnight at 4 °C, and paraffin-embedded preparations of the fly heads were then sectioned at 10 μm thickness using a HM 340E rotary microtome (Thermo Scientific Microm, Walldorf, Germany). The sections were dried at 40 °C overnight, subsequently deparaffinized with CitriSolv (Fisher Scientific, Fair Lawn, NJ, USA), and rehydrated using a series of ethanol solutions in phosphate-buffered saline (PBS). The rehydrated paraffin sections were soaked in Carson’s trichrome solution for 15 min, and the slides were gently swashed in tap water and subsequently rinsed three times in distilled water (DW). The excess water was removed with tissue paper, and the samples were mounted using Vectashield H-1000 mounting medium (Vector Laboratories, Burlingame, CA, USA).

Congo red staining was performed to detect the amyloid plaques in the *Drosophila* AD model. The paraffin-embedded sections described above were deparaffinized and then stained using NovaUltra Special Stain Kits (IHC World, Woodstock, MD, USA) according to the manufacturer’s instructions. Finally, the sections were dehydrated and mounted with mounting medium. The images were observed and captured using an EZ4 HD stereo microscope.

### Measurement of the fly eclosion rate

The pupation and emergence rates were determined according to the method of Tamura *et al.*^78^. The *elav* < *APP/BACE-1* flies were cultured from the egg stage in vials, as stated previously. Groups of 50 third instar larvae climbing on the wall were transferred into vials containing fresh media. The treated and control (0.1% DMSO only) flies were grown under the same conditions as those used for fly maintenance. The numbers of pupae and adults were determined. All treatments were repeated four times using 50 larvae per replicate.

### Lifespan assay

Groups of 20 newly emerged male *elav* < *APP/BACE-1* flies, cultured in vials as stated previously, were separately transferred to vials containing media supplemented with acacetin (100, 300, and 500 μM) every 4 days. The controls received 0.1% DMSO. The median life time (T_1/2_) was defined as the time when the survivor function equaled 50%, because median survivorship is a more reliable index than mean survival time, as described by Chakraborty and colleagues^37^. All treatments were repeated 10 times using 20 males per replicate.

### Climbing assay

The climbing activity assay was performed, as described by Wang *et al.*^33^ and Crowther *et al.*^34^. Groups of 20 male *elav* < *APP/BACE-1* flies (1, 5, 10, and 15 days old), cultured in vials as stated previously, were separately placed in an empty vial conjoined with another vial on top, and manually tapped twice. After 20 s, the flies that climbed from the bottom and crossed the 9.5 cm line were counted, and the climbing index was determined as the number of flies that climbed to the top vial relative to the total number of test flies and expressed as a percentage. The controls received 0.1% DMSO. All trials were repeated five times using 20 males per replicate.

### Feeding assay

The adult feeding assay was performed according to the methods of Wang *et al.*^33^ and Bahadorani *et al.*^79^. Groups of 15 newly emerged male *elav* < *APP/BACE-1* flies were cultured on standard media for 3 days and then starved for 20 h in vials containing three layers of Whatman no. 2 filter paper (Whatman, Maidstone, UK) soaked in DW. The flies were then transferred into vials containing media (with 0.2% Acid red) supplemented with acacetin (100, 300, and 500 μM) in 0.1% DMSO. The controls were fed with media supplemented with 0.2% Acid red and 0.1% DMSO. After 2 h of feeding, the abdomens were isolated and homogenized in 1 mL DW. After centrifugation (5000 rpm, 25 °C, 5 min), the optical density (OD) of the supernatant was measured at 505 nm, because this OD value is considered to index the amount of food intake by flies, as described by Min and Tatar^80^. All treatments were repeated three times using 15 males per replicate.

### Real-time reverse transcription-PCR analysis

Real-time qRT-PCR with SYBR Green dye was performed to determine whether acacetin affected the expression levels of the human *APP* and *BACE-1* mRNAs in the transgenic flies. The *elav* < *APP/BACE-1* and *elav* < *GFP* flies were cultured from the egg stage in vials, as stated previously. The controls received 0.1% DMSO only. The total RNA was extracted from 30 heads (30 mg) of male *elav* < *APP/BACE-1* and *elav* < *GFP* flies (20 days old) using the RNeasy Mini Kit (Qiagen, Hilden, Germany) according to the manufacturer’s instructions. The residual genomic DNA was removed using RQ1 RNase-Free DNase (Promega, Fitchburg, WI, USA), and 1 μg of the total RNA from each sample was used for complementary DNA (cDNA) synthesis with an oligo (dT) 12–18 primer (Invitrogen, Carlsbad, CA, USA) according to the protocol of the SuperScript III Reverse Transcriptase Kit. Real-time qRT-PCR was performed in 96-well plates using the StepOne Plus Real-Time PCR System (Applied Biosystems, Darmstadt, Germany). Each reaction mixture consisted of 10 μL of the SYBR Green PCR Master Mix Kit (Applied Biosystems), 2 μL of the forward and reverse primers (5 pmol each), 25 ng of the cDNA, and diethylpyrocarbonate water for a final volume of 20 μL. The oligonucleotide PCR primer pairs are listed in Table 3 and were purchased from Bioneer (Daejeon, ROK). The cycling program was an initial hold at 95 °C for 5 min, followed by 40 cycles of denaturation at 95 °C for 30 s, annealing at 62 °C for 30 s, and extension at 72 °C for 30 s. The mRNA expression levels of the target genes were normalized to the mRNA expression level of the housekeeping gene *rp 49* that encodes the *Drosophila* ribosomal protein 49, and analyzed by the 2^–ΔΔ*C*T^ method using the StepOne Software v2.3 and DataAssist Software (Applied Biosystems). The results are expressed as the means ± SEs of duplicate samples from three independent experiments.

### Western blot analysis

The *elav* < *APP/BACE-1* flies were cultured from the egg stage, as described previously. The lysates were obtained from the heads of 50 male flies (20 days old) by placing the heads in 100 μL RIPA buffer (pH 8.0) containing a 1% protease inhibitor cocktail^37^. The controls received 0.1% DMSO. The lysates were centrifuged at 14,000 rpm for 30 min at 4 °C. The protein content of the supernatant was determined using a Bradford Protein Assay kit (Sigma-Aldrich) and BSA was used as the standard. The total proteins (30 μg/sample for APP and BACE-1 detection; 70 μg/sample for Aβ detection) were mixed with an equal volume of 5× sample buffer^81^ containing 40 mM of DL-dithiothreitol, boiled for 10 min, and then loaded onto 8, 10, and 12% sodium dodecyl sulfate-polyacrylamide gels using a Mini-Protean 3 electrophoresis cell (Bio-Rad, Hercules, CA, USA) for APP, APP-CTF (APP-αCTF and APP-βCTF) and AICD, BACE-1, and Aβ detection, respectively. After electrophoresis at 110 V in 2 h, the proteins from the gels were transferred onto a polyvinyl difluoride membrane (Pall Corporation, Pensacola, FL, USA) using an electroblotting apparatus. The membrane was then blocked with 5% skim milk (BD Difco, Flanklin Lakes, NJ, USA) in PBS containing 0.1% (v/v) Tween-20 (0.1% PBS-T) at room temperature for 1 h, and further incubated overnight at 4 °C with a 1:4,000 dilution of an anti-APP C-terminal antibody to detect the cleaved APP product, a 1:1,000 dilution of anti-BACE-1, and a 1:1,000 dilution of anti-amyloid (4G8). After washing with 0.1% PBS-T three times at 10 min intervals, the membranes were further incubated for 2 h with a goat anti-rabbit IgG H&L (HRP) secondary antibody at a1:4,000 dilution for APP and BACE-1 and a goat anti-mouse lgG-HRP at a 1:5,000 dilution for β-amyloid. Finally, after washing with 0.1% PBS-T three times with a 10 min interval between washes, the membranes were developed with an ECL chemiluminescence reagent (Amersham Bioscience, Buckinghamshire, UK) and immediately exposed to a CP-PU X-ray film (AGFA, Mortsel, Belgium). The differences in protein expression were quantified using a Molecular Imager Gel Doc XR system (Bio-Rad, Hercules, CA, USA) and normalized to actin expression on the same membrane. The results are expressed as the means ± SEs of duplicate samples from three independent experiments.

## Data Analysis

The fifty percent inhibitory concentration (IC_50_) was defined as the concentration of the compound that resulted in a 50% loss of BACE-1 activity. The IC_50_ values were determined using GraphPad Prism 5.1 software (GraphPad Software, La Jolla, CA, USA). The IC_50_ values for the treatments were considered significantly different from one another when their 95% confidence limits (CLs) did not overlap. The significance of the differences between the mean values was determined using a one-way or two-way analysis of variance (ANOVA) (GraphPad Prism 5.1 software).

## Additional Information

**How to cite this article**: Wang, X. *et al.* Effects and possible mechanisms of action of acacetin on the behavior and eye morphology of *Drosophila* models of Alzheimer's disease. *Sci. Rep.* **5**, 16127; doi: 10.1038/srep16127 (2015).

## Supplementary Material

Supplementary Information

## Figures and Tables

**Table 1:** *In vitro* human BACE-1 inhibitory activity of each fraction obtained from the solvent partitioning of the methanol extract of the whole *A. rugosa* plants using a fluorescence resonance energy transfer-based enzyme assay.

Material	% inhibition at test concentration (mg/mL)		
	2	1	0.1
Methanol extract	75 ± 1.1	50 ± 1.4	24 ± 1.1
Hexane-soluble fraction	100	97 ± 1.3	47 ± 1.6
Chloroform-soluble fraction	0	0	0
Ethyl acetate-soluble fraction	20 ± 0.9	3 ± 0.9	0
Butanol-soluble fraction	0	0	0
Water-soluble fraction	0	0	0

**Table 2:** *In vitro* human BACE-1 inhibitory activity of three isolated compounds (acacetin, maslinic acid, and oleanolic acid), pure organic acacetin, and two BACE-1 inhibitors IV and epigallocatechin gallate using a fluorescence resonance energy transfer-based enzyme assay.

Compound	IC_50_, μM (95% CL)	Slope ± SE	χ^2^	*P*-value
Natural acacetin	88.5 (60.1–130.5)	0.4 ± 0.04	5.64	0.940
Pure acacetin	81.2 (59.4–111.0)	0.4 ± 0.03	5.41	0.936
Oleanolic acid	355.1 (327.3–385.4)	1.9 ± 0.12	4.07	0.989
Maslinic acid	487.6 (439.3–542.4)	1.4 ± 0.09	4.35	0.984
Epigallocatechin gallate	96.2 (72.1–128.2)	0.4 ± 0.02	3.48	0.965
BACE-1 inhibitor IV	0.079 (0.073–0.085)	1.2 ± 0.07	3.25	0.993

**Table 3:** Primers used for real-time quantitative reverse transcription polymerase chain reaction in this study.

Gene name	Forward primer and reverse primer
*rp 49*	5ʹ-CTGCTCATGCAGAACCGCGT-3ʹ
	5ʹ-GGACCGACAGCTGCTTGGCG-3ʹ
*APP*	5ʹ-GCCGTGGCATTCTTTTGGGGC-3ʹ
	5ʹ-GTGGTCAGTCCTCGGTCGGC-3ʹ
*BACE-1*	5ʹ-GCAGGGCTACTACGTGGAGA-3ʹ
	5ʹ-GTATCCACCAGGATGTTGAGC-3ʹ
*GFP*	5’-AAGTTCATCTGCACCACCG-3’
	5’-TCCTTGAAGAAGATGGTGCG-3
