# Supplementary material for: Effects and possible mechanisms of action of acacetin on the behavior and eye morphology of Drosophila models of Alzheimer’s disease
Source: Sci Rep. 2015 Nov 4;5:16127. doi: 10.1038/srep16127 (PMC4632086; doi:10.1038/srep16127)
Supplement: Supplementary Information [file srep16127-s1.pdf]

Research Article

Effects and possible mechanisms of action of acacetin on the behavior and eye morphology of *Drosophila* models of Alzheimer's disease

Xue Wang[1], Haribalan Perumalsamy[2], Hyung Wook Kwon[2], Young-Eun Na[3], and Young-Joon Ahn[1,4*]

[1]Department of Agricultural Biotechnology, Seoul National University, Seoul 151-921, Republic of Korea

[2]Research Institute of Agriculture and Life Sciences, Seoul National University, Seoul 151-921, Republic of Korea

[3]R&D Coordination Division, Rural Development Administration, Jeonju 560-500, Republic of Korea

[4]College of Plant Science & Technology, Huazhong Agricultural University, Wuhan 430070, Hubei, P.R. China

* Correspondence: yjahn@snu.ac.kr

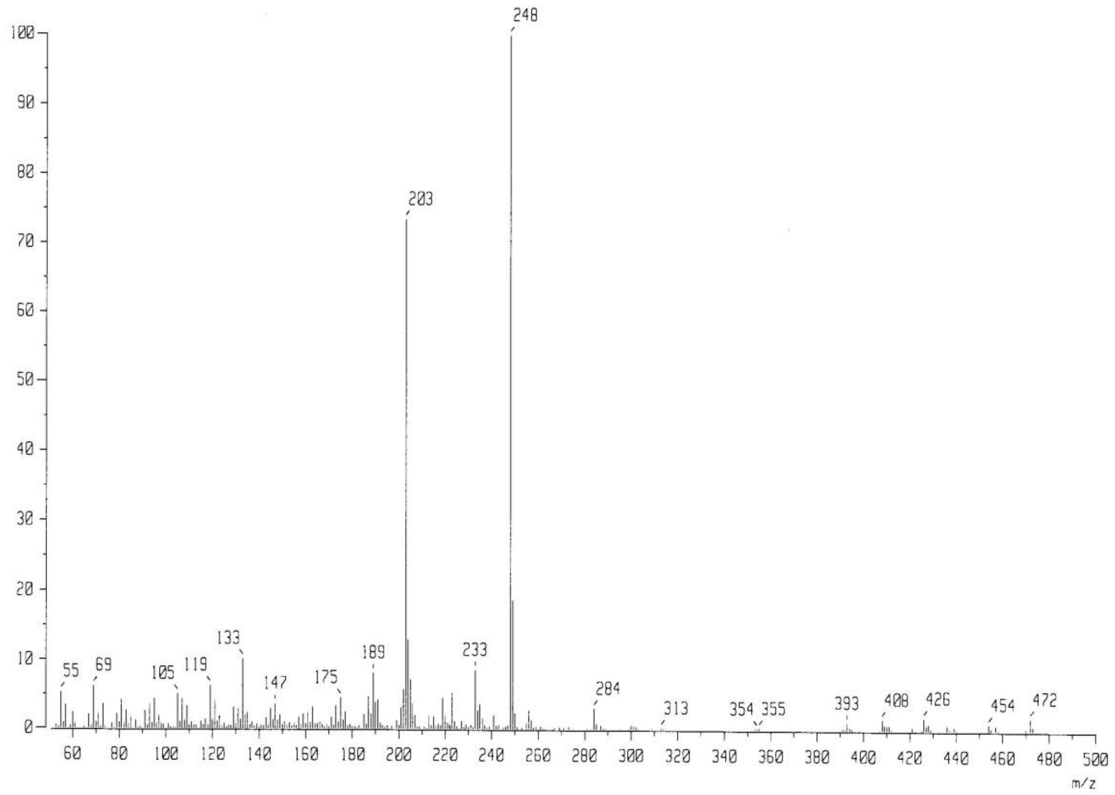

Supplementary Figure S1. EI-MS (70 ev) spectrum of maslinic acid 1.

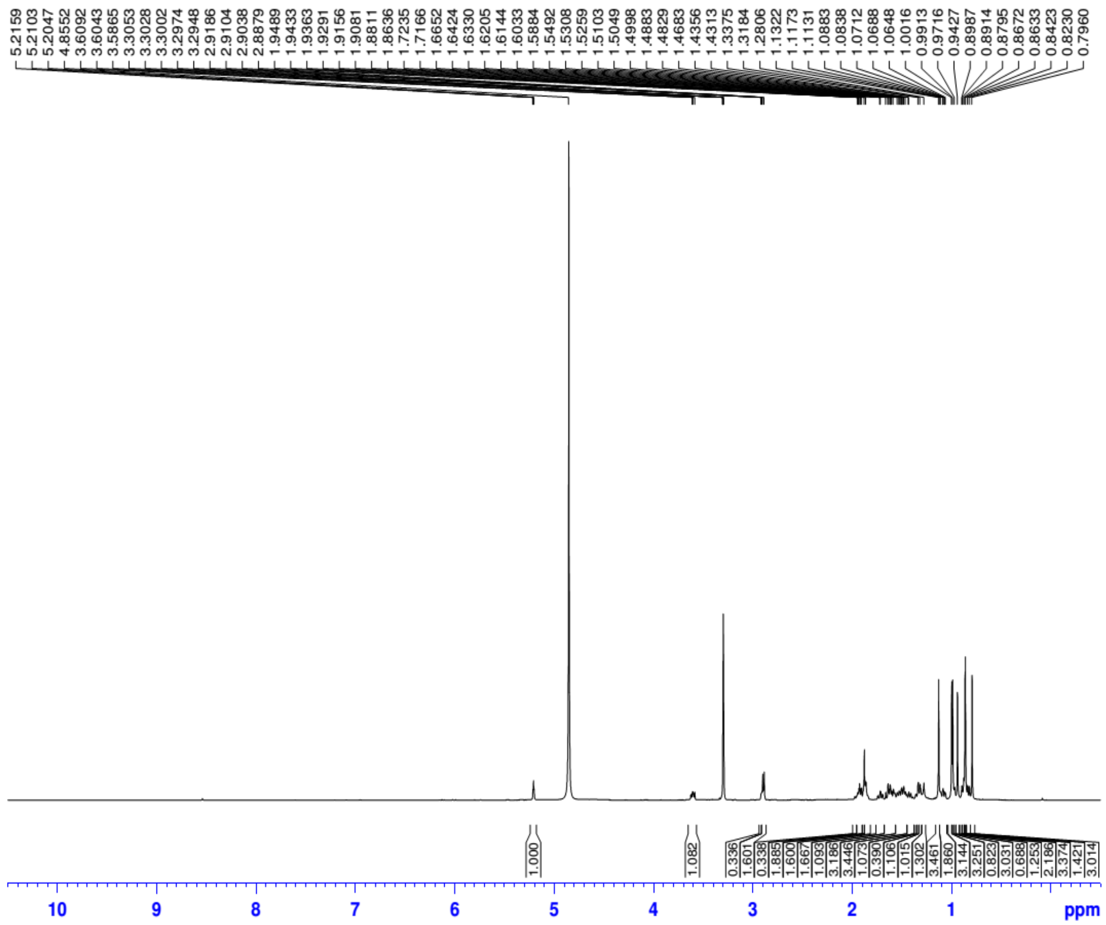

Supplementary Figure S2. ^{1}H NMR (MeOD, 600 MHz) spectrum of maslinic acid 1.

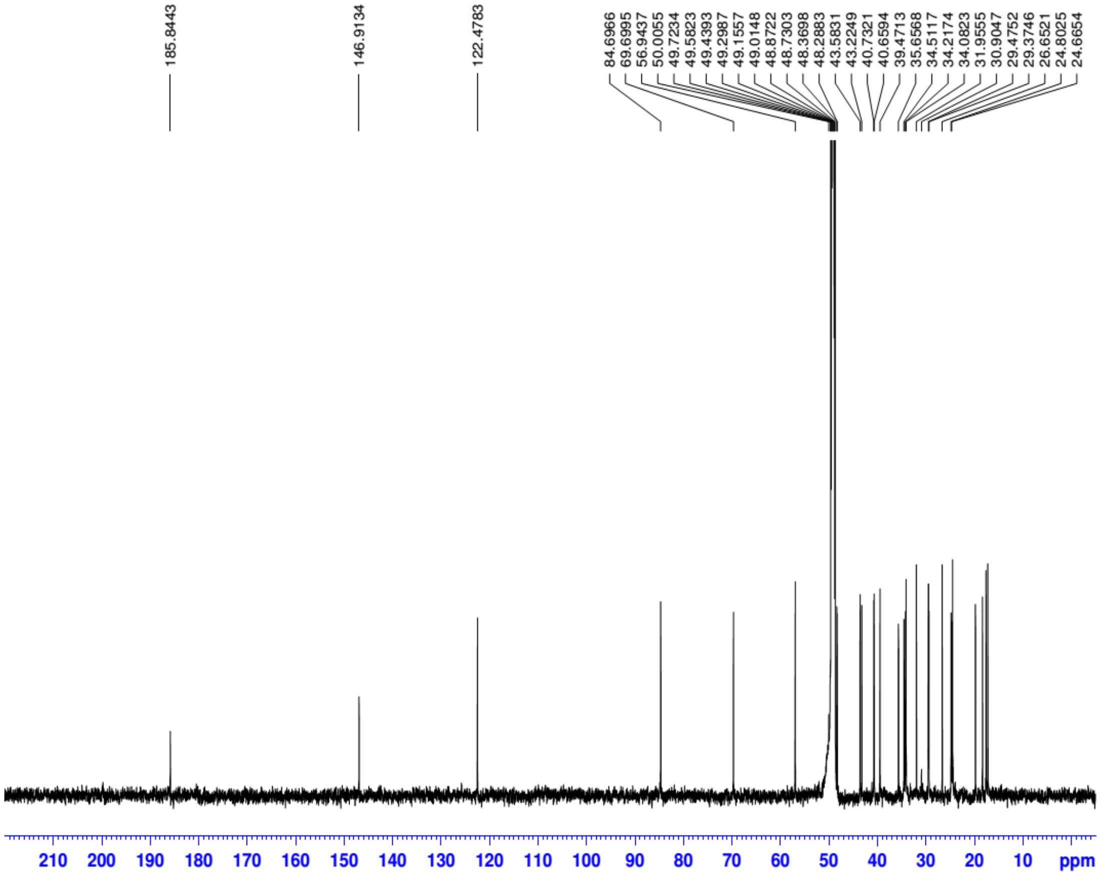

Supplementary Figure S3. ^{13}C NMR (MeOD, 150 MHz) spectrum of maslinic acid 1.

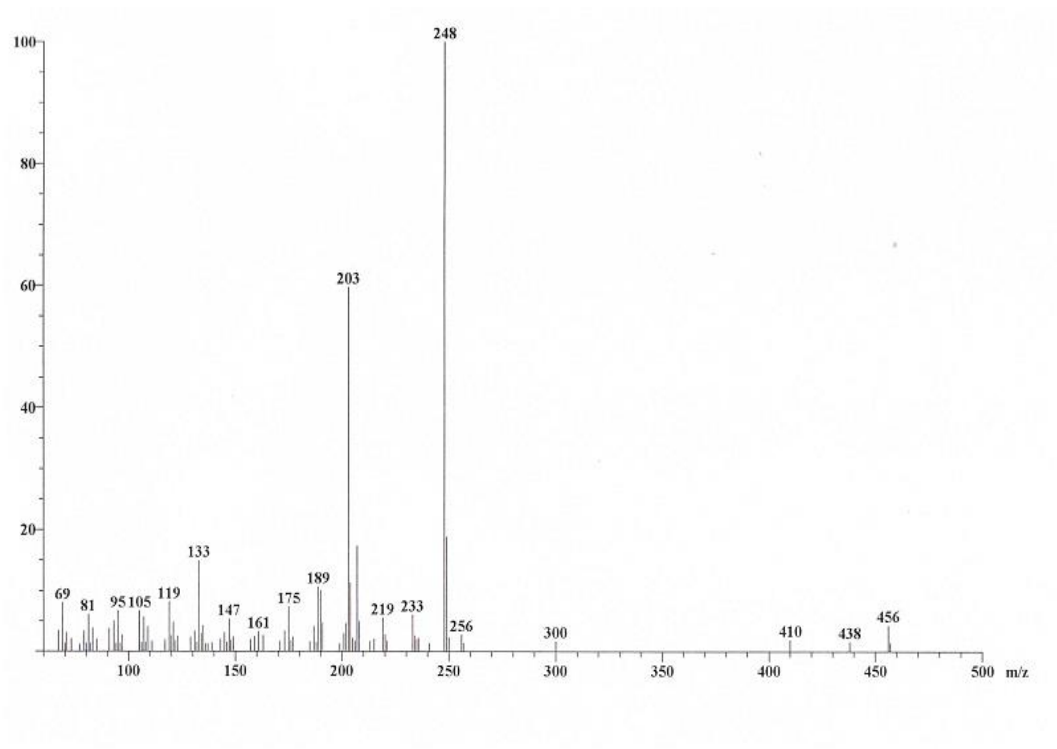

Supplementary Figure S4. EI-MS (70 eV) spectrum of oleanolic acid 2.

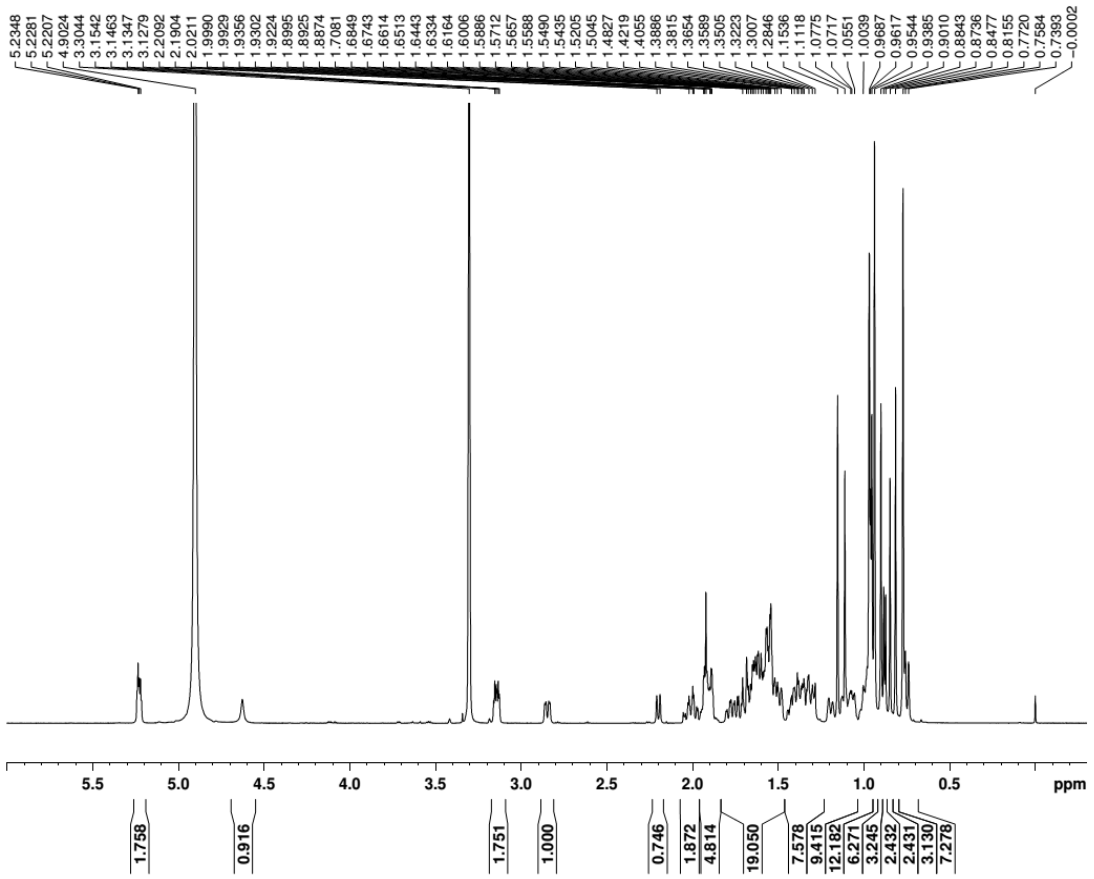

Supplementary Figure S5. ^{1}H NMR (MeOD, 600 MHz) spectrum of oleanolic acid 2.

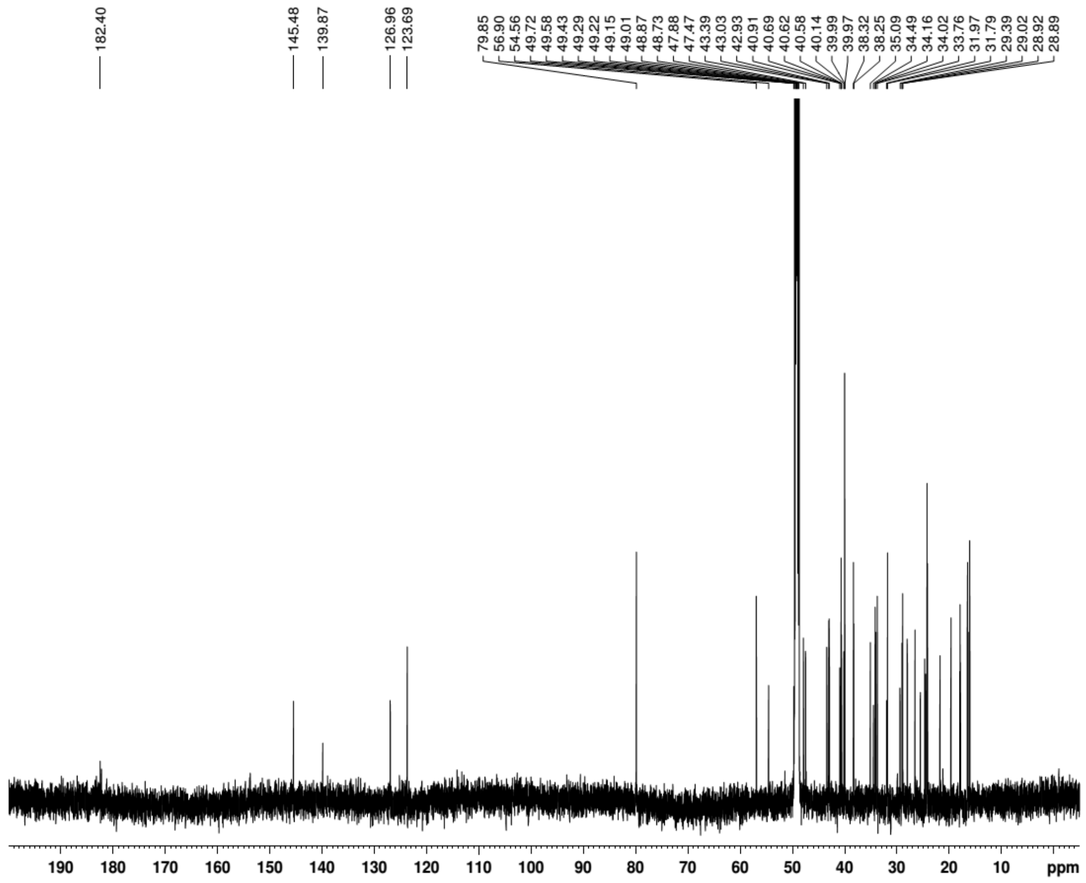

Supplementary Figure S6. ^{13}C NMR (MeOD, 150 MHz) spectrum of oleanolic acid 2.

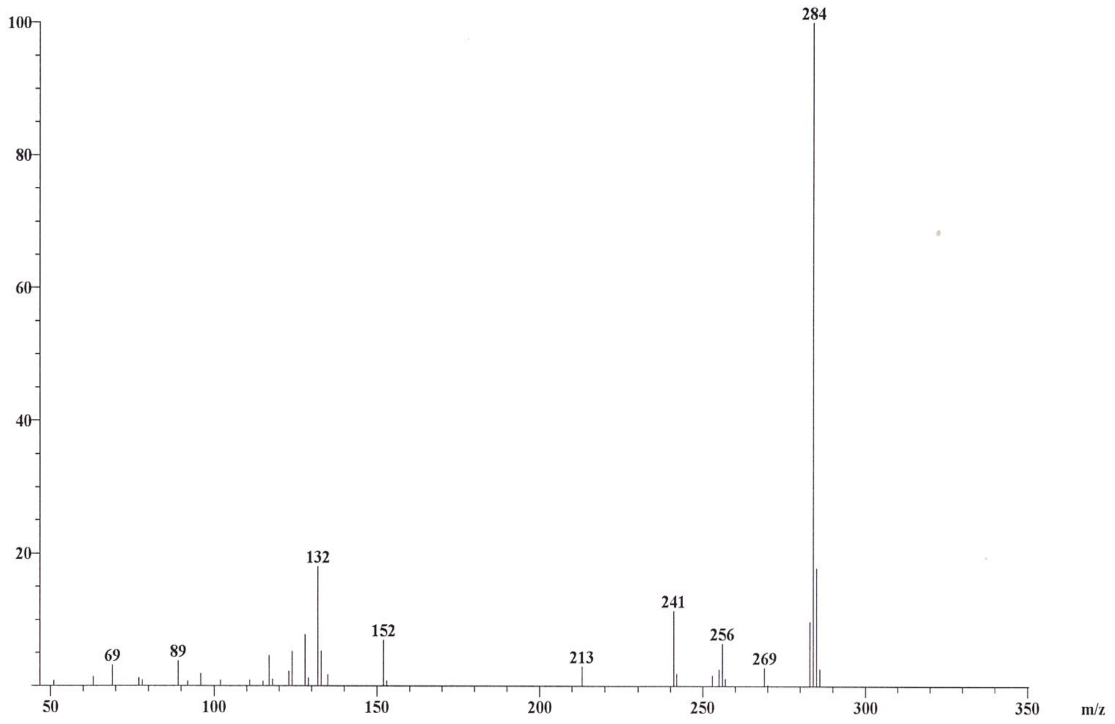

Supplementary Figure S7. EI-MS (70 eV) spectrum of acacetin 3.

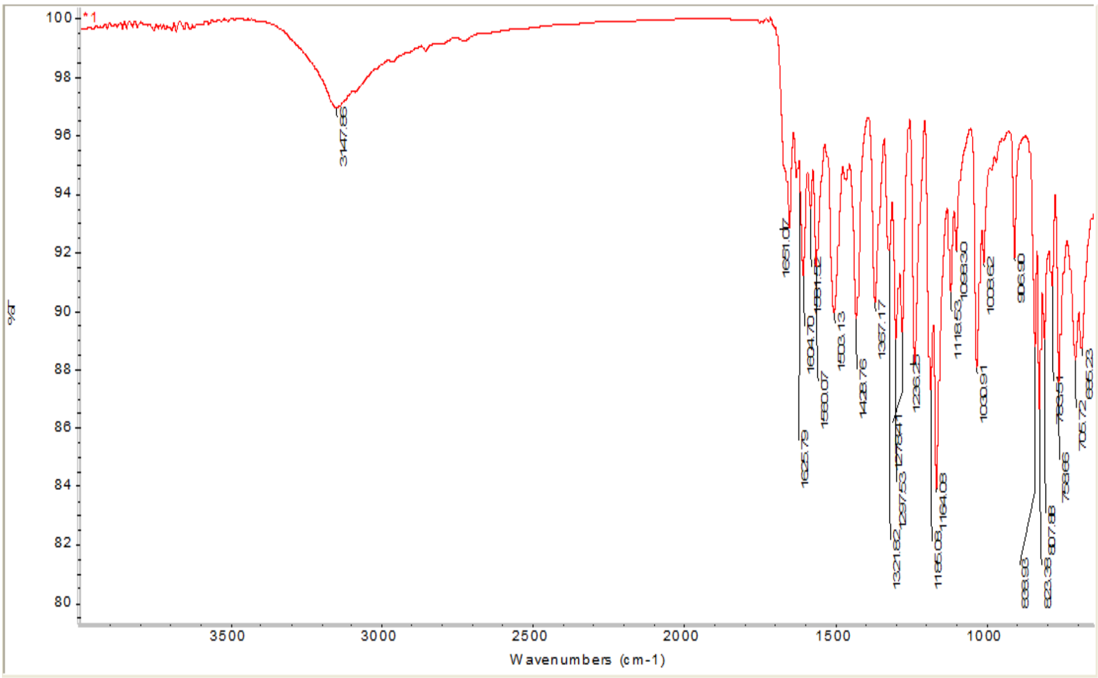

Supplementary Figure S8. FT -IR spectrum of acacetin 3.

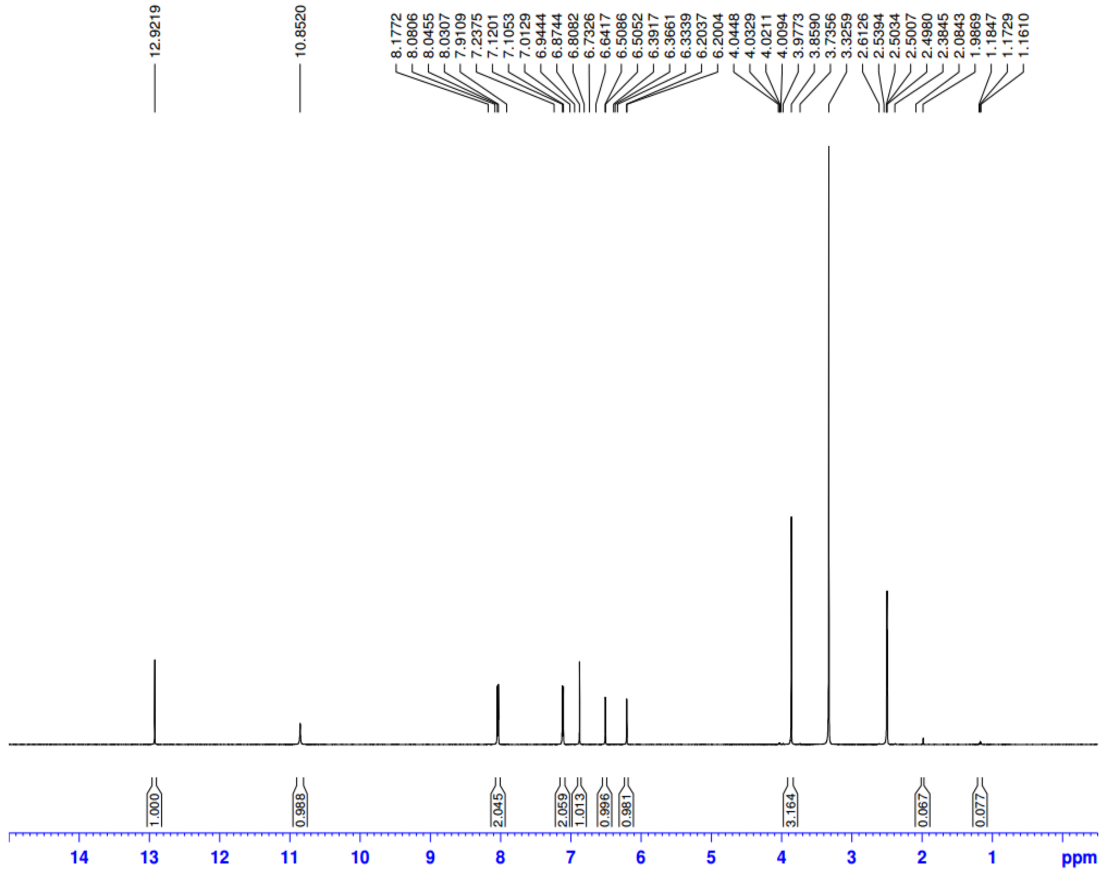

Supplementary Figure S9. **¹H NMR (DMSO-d6, 600 MHz) spectrum of acacetin 3.**

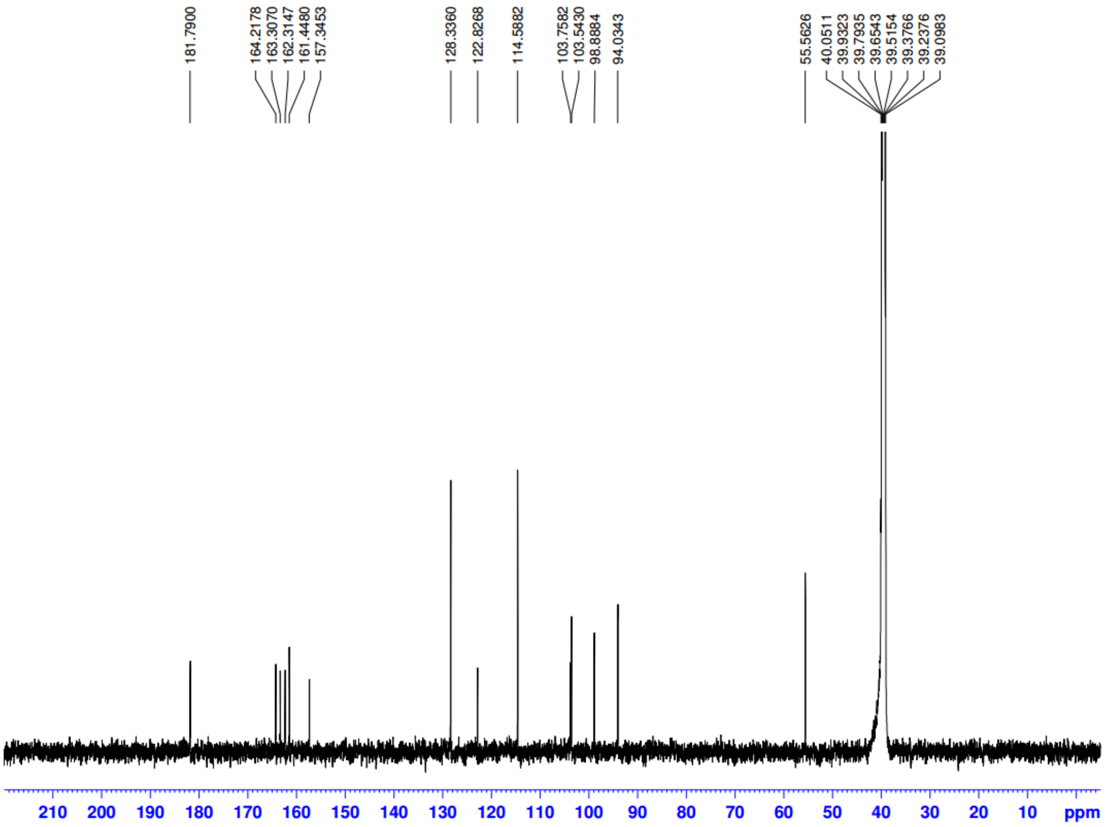

Supplementary Figure S10. ^{13}C NMR (DMSO-d6, 150 MHz) spectrum of acacetin 3.
